# The evolution of Ca^2+^-ATPases across plants with profiles in *Rhododendron* and the function of key members in alleviating high calcium stress

**DOI:** 10.1093/hr/uhag174

**Published:** 2026-04-29

**Authors:** Sulin Wen, Kui Zhou, Yi Min, Kun Yang, Chunqiong Shang, Lei Peng, Xiaowei Cai, Yuxin Leng, Luonan Shen, Lin Deng, Qingwei Zeng, Hong Zhao, Yunxing Bai, Bingxue Zhang, Manying Zhang, Qian Wang, Fuhua Fan, Xiaopeng Wen, Guang Qiao, Guijie Ding

**Affiliations:** Institute for Forest Resources & Environment of Guizhou, Guizhou Key Laboratory of Forest Cultivation in Plateau Mountain, College of Forestry, Guizhou University, Guiyang 550025, China; Key Laboratory of Plant Resource Conservation and Germplasm Innovation in Mountainous Region (Ministry of Education), Institute of Agro-Bioengineering/College of Life Science, Guizhou University, Guiyang 550025, China; Key Laboratory of Plant Resource Conservation and Germplasm Innovation in Mountainous Region (Ministry of Education), Institute of Agro-Bioengineering/College of Life Science, Guizhou University, Guiyang 550025, China; Key Laboratory of Plant Resource Conservation and Germplasm Innovation in Mountainous Region (Ministry of Education), Institute of Agro-Bioengineering/College of Life Science, Guizhou University, Guiyang 550025, China; Institute for Forest Resources & Environment of Guizhou, Guizhou Key Laboratory of Forest Cultivation in Plateau Mountain, College of Forestry, Guizhou University, Guiyang 550025, China; Key Laboratory of Plant Resource Conservation and Germplasm Innovation in Mountainous Region (Ministry of Education), Institute of Agro-Bioengineering/College of Life Science, Guizhou University, Guiyang 550025, China; Key Laboratory of Plant Resource Conservation and Germplasm Innovation in Mountainous Region (Ministry of Education), Institute of Agro-Bioengineering/College of Life Science, Guizhou University, Guiyang 550025, China; Key Laboratory of Plant Resource Conservation and Germplasm Innovation in Mountainous Region (Ministry of Education), Institute of Agro-Bioengineering/College of Life Science, Guizhou University, Guiyang 550025, China; Key Laboratory of Plant Resource Conservation and Germplasm Innovation in Mountainous Region (Ministry of Education), Institute of Agro-Bioengineering/College of Life Science, Guizhou University, Guiyang 550025, China; Key Laboratory of Plant Resource Conservation and Germplasm Innovation in Mountainous Region (Ministry of Education), Institute of Agro-Bioengineering/College of Life Science, Guizhou University, Guiyang 550025, China; Key Laboratory of Plant Resource Conservation and Germplasm Innovation in Mountainous Region (Ministry of Education), Institute of Agro-Bioengineering/College of Life Science, Guizhou University, Guiyang 550025, China; Key Laboratory of Plant Resource Conservation and Germplasm Innovation in Mountainous Region (Ministry of Education), Institute of Agro-Bioengineering/College of Life Science, Guizhou University, Guiyang 550025, China; Key Laboratory of Plant Resource Conservation and Germplasm Innovation in Mountainous Region (Ministry of Education), Institute of Agro-Bioengineering/College of Life Science, Guizhou University, Guiyang 550025, China; Key Laboratory of Plant Resource Conservation and Germplasm Innovation in Mountainous Region (Ministry of Education), Institute of Agro-Bioengineering/College of Life Science, Guizhou University, Guiyang 550025, China; College of Ecological Engineering, Guizhou University of Engineering Science, Bijie, Guizhou 551700, China; Institute for Forest Resources & Environment of Guizhou, Guizhou Key Laboratory of Forest Cultivation in Plateau Mountain, College of Forestry, Guizhou University, Guiyang 550025, China; Yazhouwan National Laboratory, Sanya, Hainan 572025, China; Key Laboratory of Plant Resource Conservation and Germplasm Innovation in Mountainous Region (Ministry of Education), Institute of Agro-Bioengineering/College of Life Science, Guizhou University, Guiyang 550025, China; Key Laboratory of Plant Resource Conservation and Germplasm Innovation in Mountainous Region (Ministry of Education), Institute of Agro-Bioengineering/College of Life Science, Guizhou University, Guiyang 550025, China; Institute for Forest Resources & Environment of Guizhou, Guizhou Key Laboratory of Forest Cultivation in Plateau Mountain, College of Forestry, Guizhou University, Guiyang 550025, China; Key Laboratory of Plant Resource Conservation and Germplasm Innovation in Mountainous Region (Ministry of Education), Institute of Agro-Bioengineering/College of Life Science, Guizhou University, Guiyang 550025, China; Key Laboratory of Plant Resource Conservation and Germplasm Innovation in Mountainous Region (Ministry of Education), Institute of Agro-Bioengineering/College of Life Science, Guizhou University, Guiyang 550025, China; Institute for Forest Resources & Environment of Guizhou, Guizhou Key Laboratory of Forest Cultivation in Plateau Mountain, College of Forestry, Guizhou University, Guiyang 550025, China

## Abstract

Ca^2+^-ATPase (CAP) is a key Ca^2+^ efflux protein in plants. Our previous research suggests that CAPs may play a crucial role in the adaptation of rhododendrons to high calcium environments. However, the evolution, variation, characteristic expression, and subfunctionalization of this gene family in *Rhododendron* remain unknown. Through the analysis of pan-genomes and pan-transcriptomes, we elucidated the systematic evolution of CAPs in plants, as well as their characteristic expression patterns in *Rhododendron*. During the evolutionary process from lower to higher plants, CAPs can be divided into six clades and exhibit structural conservation. CAPs have emerged and differentiated in lower plants such as algae, and they have undergone significant amplification in Eudicots plants like rhododendrons. Three *Rhododendron* species (*Rhododendron bailiense*, *R. delavayi*, and *R. irroratum*) located in the karst province of Guizhou in Southwest China exhibit the highest copies of CAPs, suggesting a strong association between CAP copy number variation and habitat, particularly in high calcium environments. Through multiple transcriptome analyses, we revealed that CAPs are induced under various environmental/developmental conditions (e.g. karst environments, high altitude, early flower development, hormones, etc.). Co-expression network analysis highlighted key members of calcineurin B-like protein (CBL) and CBL-interacting protein kinases (CIPK) that are associated with the high expression of CAPs. Experimental validation demonstrated that CAPb1 and CAPd1 significantly alleviate high calcium stress, and the CAPb1-CIPK1-CBL1 and CAPd1-CIPK2-CBL1 modules can further enhance the alleviation. These findings provide new insights into the evolution, characteristic expression, and function of CAPs, as well as new perspectives on the high calcium adaptability of rhododendrons.

## Introduction

The genus *Rhododendron* in the family Ericaceae is the largest genus of woody plants in the Northern Hemisphere, comprising over 1000 wild species and 30 000 cultivated varieties [1–3]. Due to the rich ecological types, genetic diversity, and habitats of *Rhododendron*, it has become an ideal material for studying environmental adaptive evolution and may potentially serve as a model plant for such research [4, 5]. Our team has recently completed a high-quality genome assembly of the rare karst-growing species *Rhododendron bailiense*, which grows in Guizhou, southwestern China [2]. This genome assembly reveals that a core feature of *R. bailiense* is the presence of abundant Ca^2+^-ATPase (CAP) genes. These genes primarily function in the efflux of Ca^2+^ [6, 7] and may play a crucial role in the environmental adaptability related to karst and high calcium of various plant species, which is one of the factors contributing to the widespread distribution of *Rhododendron* in Southwest China. However, current research on CAPs in plants remains relatively scarce, especially in terms of evolutionary aspects.

Ca^2+^ signaling is a crucial transducer in plant development and stress responses [8]. These Ca^2+^ signals are generated and transmitted by Ca^2+^ transport systems and are decoded by various calcium-binding signaling proteins [6, 7]. The cytosolic Ca^2+^ concentration is regulated by Ca^2+^ influx and efflux transporters, including pumps, calcium channels, and exchangers. The cytosolic efflux transporters consist of low-affinity/high-capacity Ca^2+^/H^+^ antiporters (CAXs) and high-affinity/low-capacity Ca^2+^-ATPases (CAPs). CAPs can be categorized into endoplasmic reticulum (ER)-type Ca^2+^-ATPase (ECA or P2A ATPase) or autoinhibited Ca^2+^ ATPase (ACAs or P2B ATPase) [9, 10]. The activity of autoinhibited CAPs is self-repressive, mediated by an N-terminal auto-inhibitory domain (AD), but this inhibition can be modulated and alleviated by Ca^2+^ or calmodulin [7]. Therefore, the Ca^2+^ efflux system may be influenced by environmental factors and regulate intracellular Ca^2+^ concentration. Research in *Arabidopsis* indicates that CAPs broadly impact plant responses to biotic/abiotic stress and growth development [10, 11]. Despite the numerous studies conducted on Ca^2+^ influx in plants, research on Ca^2+^ efflux, homeostatic mechanisms, and high calcium tolerance remains scarce, particularly concerning horticultural and forestry plants. Additionally, studies on the evolutionary history of related genes in plants are also limited.

Rhododendrons exhibit strong adaptability and are widely distributed. East Asia boasts the richest species diversity of *Rhododendron* plants globally, far surpassing that of Europe and North America [1, 5, 12]. The phenomenon of evolutionary radiation in rhododendrons is particularly pronounced within the angiosperms. Southwest China is home to the largest number of *Rhododendron* species in the world [5], and the complex geographical environment of this region has fostered many endemic species adapted to local conditions [1]. The rare species known as *R. bailiense*, located in the Baili Rhododendron Nature Reserve (BRNR) in Guizhou, serves as a typical representative [13]. Its habitat is unique, characterized by extreme karst topography, and it may be subjected to severe stresses related to karst-induced drought, high temperature, and high calcium stress [2]. High calcium levels are one of the most significant geographical features of karst regions, and plants must adopt appropriate genetic strategies to cope with the extremely high concentrations of Ca^2+^; otherwise, it may lead to Ca^2+^ overload and toxicity [14, 15]. The extracellular concentration of Ca^2+^ is typically over 10 000 times that of the cytosolic concentration [15]. To prevent the cytotoxicity of Ca^2+^, a low concentration of Ca^2+^ in the cytosol (approximately 100 nM) must be maintained. Karst environments may be an ideal type for studying the adaptation of forest plants to high calcium levels. Furthermore, karst landforms cover approximately 33% of China’s land area and about 15% of the global total area, primarily distributed in the southwestern region of China [2]. Therefore, enhancing research on high calcium tolerance associated with karst is necessary and may apply the theoretical knowledge gained to the screening and creation of resilient tree species germplasm and the governance actions for karst stony desertification.

With the rapid development of third-generation sequencing technology [16], many species, including rhododendrons, have completed high-quality genome sequencing assemblies [17]. Currently, approximately 20 species of rhododendrons have published high-quality genomes, most of which have emerged in the past 5 years [3, 4, 18–22]. In 2017, the first *Rhododendron* genome (*Rhododendron delavayi*) was published [23]. In 2024, our team published the high-quality genome of the rare species, *R. bailiense*, which grows in the karst region of Guizhou [2, 24]. Interestingly, the genome of *R. bailiense* possesses some distinct features that may determine its adaptability to the karst environment [25]. One of the most significant genes amplified during the evolutionary process is the Ca^2+^ transport protein gene, with ‘CAP’ being a prominent representative and another important representative being the ‘ankyrin repeat gene’ (a type of passive Ca^2+^ transporter) [2]. The differences in gene family sizes and subfamilies among species contain critical information that may help us understand the evolutionary history and environmental adaptations of plants [26, 27]. Although copy number variations (CNVs) have been widely identified in pan-genome studies, most evolutionary and systematic genome-wide identification studies of gene families are still based on a single reference genome [28, 29], with little attention paid to the pan-genomic analysis of specific gene families, including CAPs.

Based on the recent publication of dozens of plant genomes, we analyzed the evolutionary history of CAPs in plant evolution. We also conducted a pan-genome analysis of CAPs across *Rhododendron* genomes and explored their potential associations with the diverse habitats of *Rhododendron* species. We applied self-sequenced and publicly available transcriptomic data of various *Rhododendron* species to explore the expression patterns of CAPs under different environmental conditions. Additionally, we elucidated the co-expression gene network of CAPs and identified potential key genes that play regulatory or synergistic roles. We experimentally verified that two key members of the CAPs have the function of alleviating high calcium stress, and we validated their synergistic functions with regulatory genes. Our research enhances the understanding of the evolution, expression, and functionality of CAPs, providing new insights into the evolutionary adaptability of *Rhododendron* species to high calcium environments. Furthermore, it may offer theoretical references for the adaptive breeding and improvement of *Rhododendron* and other plants, which may further contribute to the management of karst-fragile habitats and ecological sustainability.

## Results and discussion

### Phylogenetic relationships of Ca^2+^-ATPases in plants

**Figure 1:**
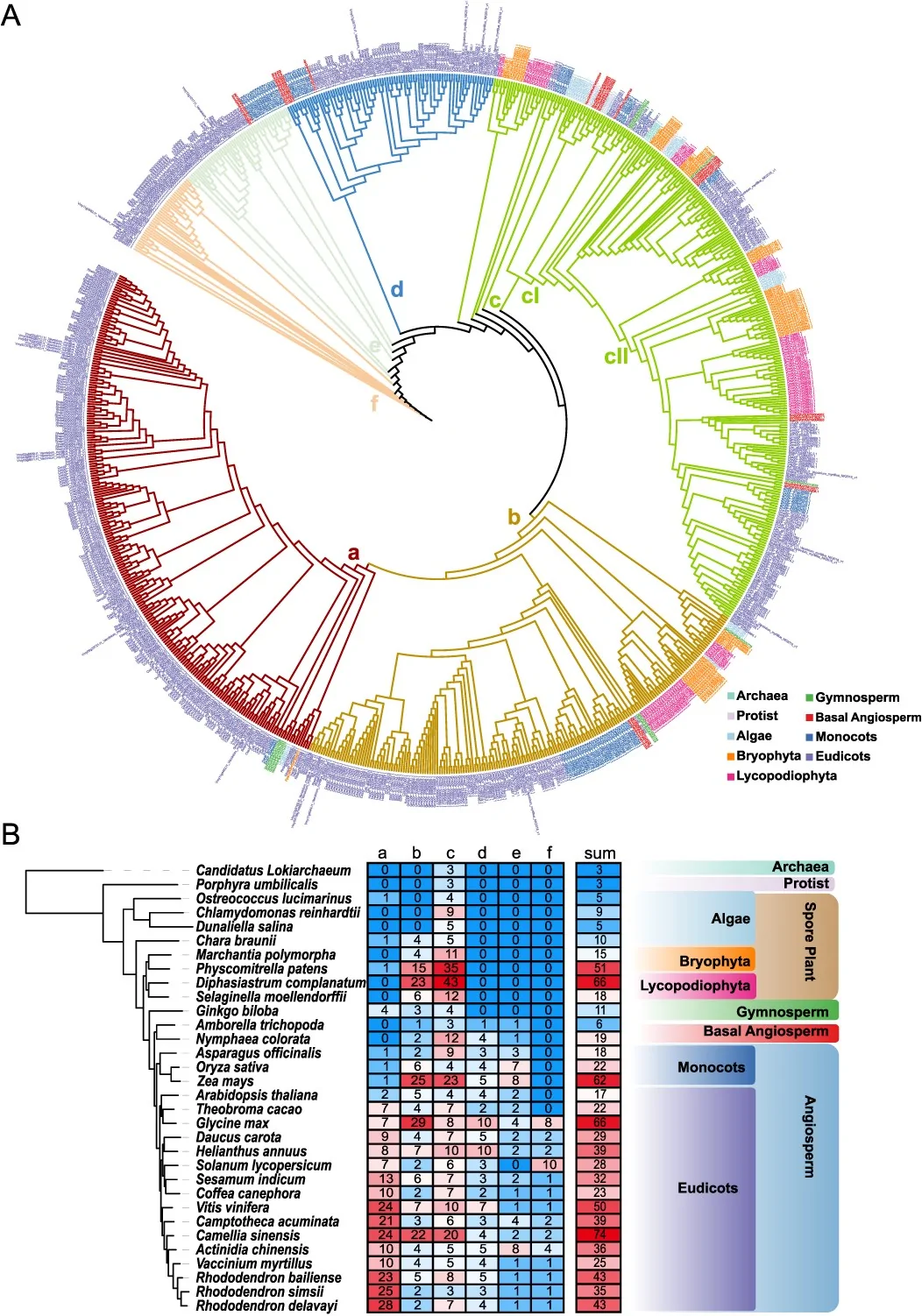
Phylogenetic relationships of the CAP gene family in plants, Protist, and Archaea. (A) Phylogenetic tree of the CAP gene family in plants, Protist, and Archaea. CAPs were divided into six clades (CAP-a to CAP-f). Among them, the larger CAP-c has two major subclades (cI and cII). The prefix labels correspond to the following species: ‘Lok’, *Candidatus Lokiarchaeum*; ‘Pum’, *P. umbilicalis*; ‘Olu’, *O. lucimarinus*; ‘Cre’, *Chlamydomonas reinhardtii*; ‘Dsa’, *Dunaliella salina*; ‘Cbr’, *C. braunii*; ‘Mp’, *M. polymorpha*; ‘Ppa’, *Physcomitrella patens*; ‘Dco’, *D. complanatum*; ‘Smo’, *Selaginella moellendorffii*; ‘Gbi’, *G. biloba*; ‘Atr’, *Amborella trichopoda*; ‘Nco’, *Nymphaea colorata*; ‘Os’, *O. sativa*; ‘Zm’, *Zea mays*; ‘At’, *A. thaliana*; ‘Cco’, *Theobroma cacao*; ‘Gm’, *Glycine max*; ‘DCAR’, *Daucus carota*; ‘Ha’, *Helianthus annuus*; ‘Soly’, *Solanum lycopersicum*; ‘Si’, *Sesamum indicum*; ‘Cca’, *Coffea canephora*; ‘Vv’, *Vitis vinifera*; ‘Cac’, *Camptotheca acuminata*; ‘Css’, *Camellia sinensis*; ‘Ac’, *Actinidia chinensis*; ‘Vmy’, *Vaccinium myrtillus*; ‘Rb’, *R. bailiense*; ‘Rs’, *R. simsii*; ‘Rd’, *R. delavayi*. The model used for constructing the phylogenetic tree is JTT + F + R6. Using IQtree with three different models (JTT + R10, JTT + R5, and LG + I) can also produce the overall structure of the six clades of CAPs. (B) The number of copies of each clade member possessed by each species.

To understand the variations of CAPs in the evolutionary history of plants, we collected genomes from 31 species representing different stages of plant evolution for phylogenetic analysis. Typical CAPs exhibit distinct structural characteristics such as transmembrane domains and ATP-binding domains. Based on these characteristic features of CAPs, we identified CAP genes in each plant genome and constructed a phylogenetic tree (Fig. 1A), while also counting the number of members in each clade for each species (Fig. 1B). This phylogenetic tree encompasses representative plants from Archaea (e.g. *Candidatus lokiarchaeum*), Protists (e.g. *Porphyra umbilicalis*), Algae (e.g. *Ostreococcus lucimarinus*), Bryophyta (e.g. *Marchantia polymorpha*), Lycopodiophyta (e.g. *Diphasiastrum complanatum*), Gymnosperms (e.g. *Ginkgo biloba*), Monocots (e.g. *Oryza sativa*), and Eudicots (e.g. *Arabidopsis thaliana*), totaling 906 CAP genes. The CAPs are classified into six major clades, labeled a–f. Among these, CAP-d, CAP-e, and CAP-f belong to early clades, CAP-c is a transitional clade, while CAP-a and CAP-b represent late clades. CAP-c includes many members and features two main subclades (cI and cII), encompassing all species from Archaea to Eudicots, illustrating the evolutionary history of CAPs from lower plants to higher plants. CAP-a primarily includes members of Eudicots, with members of the *Rhododendron* family covering the end of the phylogenetic tree (Fig. 1A and B). We found that early clades (CAP-d to CAP-f) are predominantly composed of members of higher plants, while members of lower plants are absent, suggesting that members of CAP-d to CAP-f may have been lost during the evolutionary process in lower plants. From CAP-b to CAP-c, a significant number of members from Algae, Bryophyta, and Lycopodiophyta are included (Fig. 1B), indicating that CAPs have diversified and undergone substantial amplification in lower plants. Whole genome duplication and tandem duplication may be responsible for the extensive amplification of CAPs [27, 30]. For the early species *Chara braunii* in plant evolutionary history, it includes members from CAP-a to CAP-c (Fig. 1A and B), indicating that three clades of CAPs have emerged in lower plants. Through an analysis of the CAPs domains, except for a few truncated members, most CAP members exhibit relatively conserved structural characteristics, with lengths and core domains (such as Cation_ATPase and Hydrolase) being similar among members (Supplementary Fig. S1). Additionally, features at the N-terminus may be key factors in distinguishing the clades of CAPs.

Research related to the functions of CAPs has already been conducted [11, 31], but studies on the evolutionary systematics of this gene family remain scarce. In this study, we present the systematic evolutionary relationships of CAPs within the evolutionary history of plants. The three-dimensional structures of CAPs in both animals and plants exhibit a high degree of conservation, suggesting that CAPs originated early in the ancestral lineage of life and have maintained a conserved state throughout biological evolution [2, 32]. The presence of ancestral CAPs in both Archaea and green algae indicates a long evolutionary history of CAPs and highlights the ancestral requirement for Ca^2+^ efflux. In summary, these findings primarily reveal that CAPs emerged and diversified early in plant evolution, as well as the conserved structural features among CAP members.

### Pan-genome analysis of CAPs in *Rhododendron*

**Figure 2:**
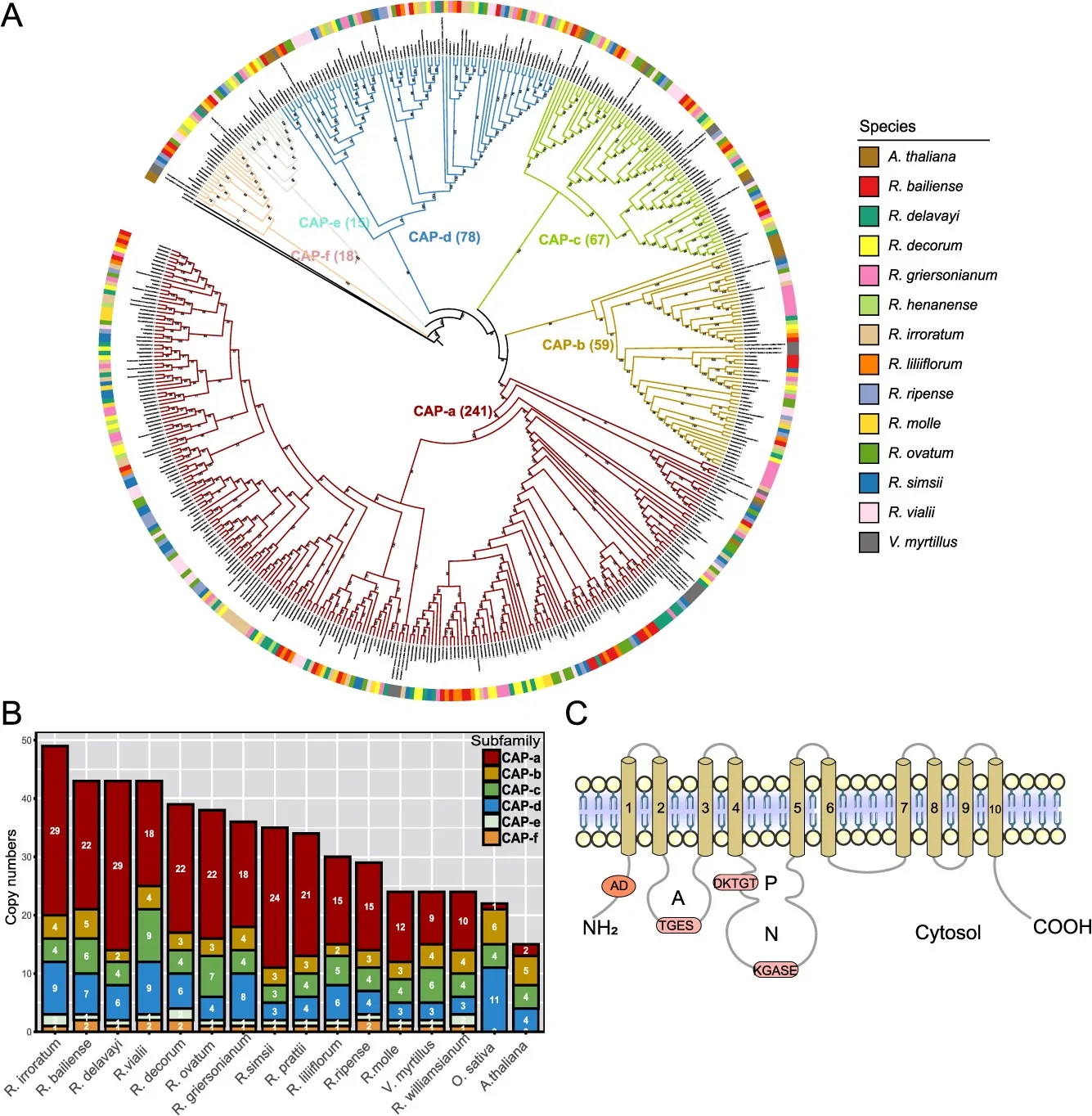
Phylogenetic relationships and copy number analysis of Ca^2+^-ATPases (CAPs) at the pan-genome level in rhododendrons, *Arabidopsis*, and rice. (A) Phylogenetic tree of CAPs in *Rhododendron* species, *Arabidopsis*, and rice. The model used for constructing the phylogenetic tree is JTT + R10. (B) Comparison of the copy numbers of CAP-a to CAP-f subfamilies across species and total CAPs copy numbers. Overall, the species of *Rhododendron* in high-altitude regions (primarily belonging to the subgenera *Hymenanthes* and *Rhododendron*) possess a higher copy number of CAPs compared to those in low-altitude regions (mainly represented by the subgenera *Tsutsusi* and *Pentanthera*). (C) Schematic diagram of the structure of autoinhibited CAPs. The typical length of CAP protein is approximately 1000 amino acids. A normal autoinhibited CAPs is a single polypeptide comprising ten transmembrane (TM) domains and a cytoplasmic head that includes critical functional regions. The large loop between TM4 and TM5 contains the nucleotide-binding domain (N-domain), characterized by the conserved KGASE motif responsible for ATP binding, as well as the phosphorylation domain (P-domain), which features the DKTGT motif. Additionally, the small cytosolic loop between TM2 and TM3 harbors the TGES motif, forming part of the actuator domain (A-domain). Together, the N-, P-, and A-domains play an integral role in facilitating ATP hydrolysis.

Environmental factors may shape the amplification and contraction of CAPs in plants, particularly for woody plants like *Rhododendron*. To further investigate the phylogenetic history of CAPs in the genus *Rhododendron*, the genomes of 12 high-quality *Rhododendron* species were collected, primarily consisting of wild species from various regions. The re-annotation of these genomes allowed us to identify the catalog of CAP members (Supplementary Data Set S1), and these members were verified for accuracy through careful examination. This process revealed more CAP members in *Rhododendron* than the previous study (based on orthogroups identification methods) [2]. The CAPs in *Rhododendron* can also be divided into six main clades (Fig. 2A). Among these, CAP-a has the highest number of classified members, totaling 241. In contrast, CAP-e has the fewest members, with only 15. The distribution of CAP families across different species was further analyzed (Fig. 2B). Notably, the copy number of CAPs exhibits a stepwise distribution from high to low among different *Rhododendron* species, suggesting a persistent change in CAPs during the evolutionary process of *Rhododendron*. All *Rhododendron* species possess members from the CAP-a to CAP-f families, but their distribution varies (Fig. 2B). For *Rhododendron* species, the CAP-a subfamily has the most members and is the most frequently duplicated. Each species of *Rhododendron* contains more than one member from the CAP-b to CAP-d groups. Notably, the copy numbers of CAPs in *Arabidopsis* and rice, especially for CAP-a, are significantly lower than those of the members in rhododendrons, suggesting that the environment in which rhododendrons exist has led to the specific evolution of CAPs, differing from the model plants *Arabidopsis* and rice. For CAPs, which are membrane transport proteins, their typical structure is illustrated in Fig. 2C. Sequence analysis indicates that the CAP motifs in *Rhododendron* are relatively conserved, with most motif distribution patterns similar to those of other subfamily members (Supplementary Note S1; Supplementary Fig. S2), suggesting functional similarities. This is also consistent with the results in the previous section. Functional enrichment analysis reveals that the CAPs of different subfamilies exhibit distinct subfunctionalities, and there are also differences in subcellular localization (Supplementary Note S2; Supplementary Figs S3 and S4). For example, CAP-b is primarily located in the plasma membrane, while CAP-d is mainly found in the endoplasmic reticulum.

Previous research on CAPs has primarily focused on monomers and individual reference genomes, with limited attention given to their systematics and evolution [6, 7]. Furthermore, traditional studies on CAPs have predominantly centered on *Arabidopsis*, while research on nonmodel plants remains scarce [7]. Owing to the recent extensive publication of the *Rhododendron* genomes, the copy number variations, evolution, and function of CAPs at the pan-genome level in rhododendrons were summarized. As a wild woody plant, *Rhododendron* has a greater advantage over the model herbaceous plant *Arabidopsis* in terms of long-term ecological conservation [33]. Through the exploration of rhododendrons, we also transcend the limitations of laboratory settings [34], investigating the genotype in wild plants and their interactions with the environment, while incorporating a richer plant diversity. Furthermore, our results suggest that CAPs in rhododendrons have a broad role in responding to environmental stimuli and regulating growth and development. Importantly, our findings demonstrate a stepwise variation in the copy number of CAPs among different *Rhododendron* species (Fig. 2B), indicating a continuous change in CAPs throughout the evolutionary process of rhododendrons. This continuous stepwise distribution leads us to hypothesize that the copy number of CAPs may be associated with the subgenus and geographical distribution of rhododendrons, potentially linked to the different environments in which various *Rhododendron* species are found. This issue will be explored in the subsequent section.

### The relationship between the habitat of rhododendrons and CAPs

**Figure 3:**
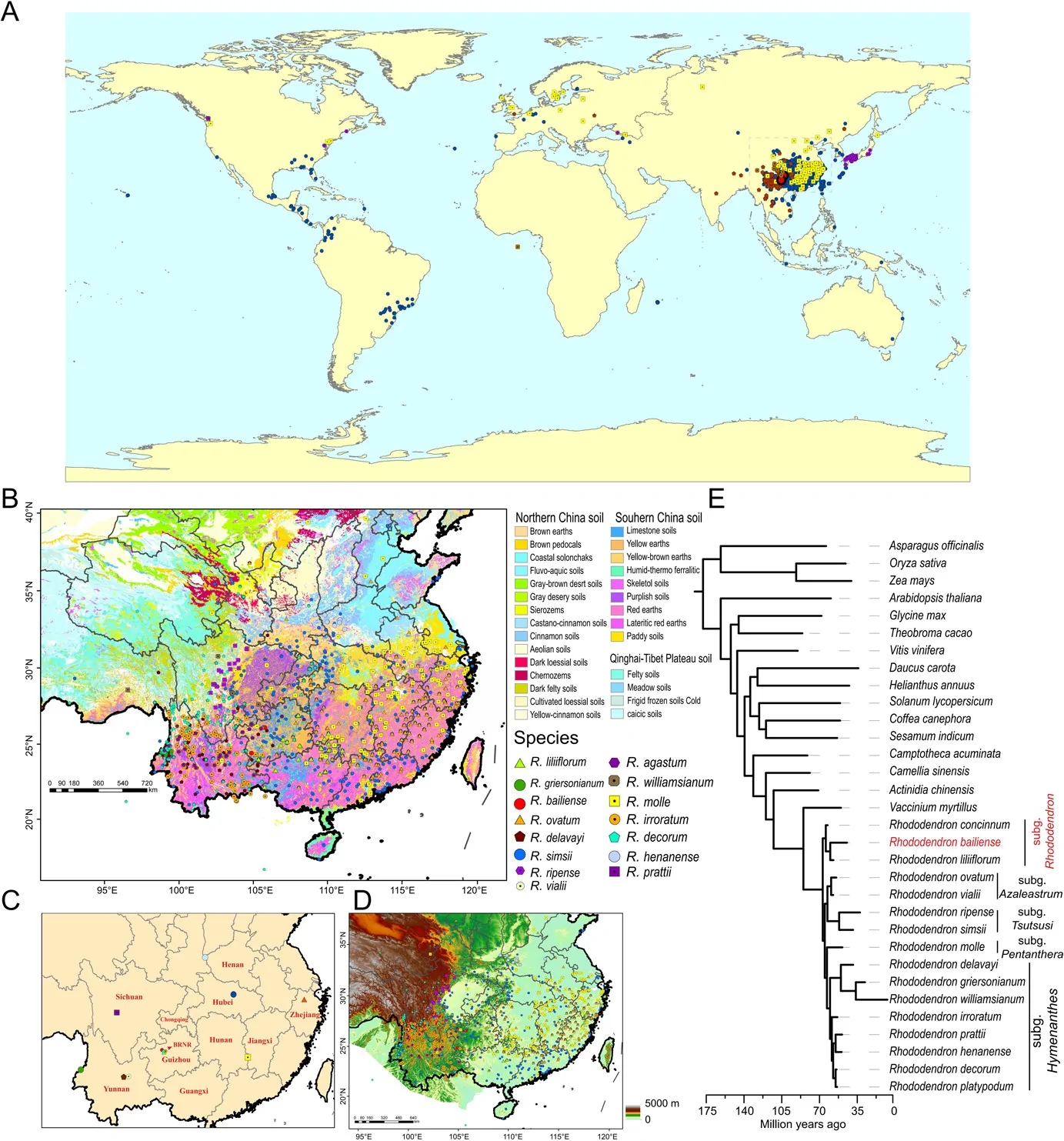
Geographical distribution of *Rhododendron* species. (A) The global distribution of *Rhododendron* species involved in this study. (B) The distribution of *Rhododendron* species in southern and some northern regions of China, along with different soil types. Panel (B) corresponds to the position of the gray dashed box in panel (A). (C) The provinces from which the *Rhododendron* individuals used for genomic sequencing were collected. The names of the provinces where rhododendrons were collected, along with the names of surrounding provinces, are marked in red. Among them, the Baili Rhododendron Nature Reserve (BRNR) located in Guizhou has provided 4 *Rhododendron* samples for genomic research. (D) A map showing the elevation distribution of China’s topography and the distribution of rhododendrons. (E) The phylogenetic tree and chronogram of species demonstrate the evolutionary history of rhododendrons. The subgenera *Hymenanthes* and *Rhododendron* are primarily distributed in the western high-altitude regions, while the subgenera *Tsutsusi*, *Pentanthera*, and *Azaleastrum* are mainly found in the eastern low-altitude areas.

The global distribution of the main species of *Rhododendron* that have been genome sequenced to date was analyzed (Fig. 3A). The map indicates that the sequenced *Rhododendron* species are predominantly concentrated in southern China. Meanwhile, *Rhododendron simsii* is the most widely distributed species of *Rhododendron* globally, found across Europe, North America, and South America. Common horticultural species such as *Rhododendron molle* and *R. delavayi* are also present in Europe. For the *Rhododendron* species distributed in southern China, their distribution map was created (Fig. 3B–D). The collection sites for the *Rhododendron* genome samples used in this study are shown in Fig. 3C. The *Rhododendron* genome samples were sourced from different provinces in China, including Guizhou, Yunnan, Sichuan, Hubei, Hunan, Zhejiang, and Henan. The most samples were collected from BRNR in Guizhou, including *R. bailiense*, *Rhododendron liliiflorum*, *Rhododendron irroratum*, and *Rhododendron decorum* [21]. The geographical topography of China presents a pattern of high elevation in the west and low elevation in the east, with the eastern region primarily consisting of plains and hills, while the southwestern region is mountainous (Fig. 3D). The southwestern region is also the area with the richest diversity of *Rhododendron* species in China [1, 12]. The boundaries between provinces in China are distinct, often demarcated by mountains and rivers, which leads to the presence of unique soil types or terrains within the provinces (e.g. Guizhou) (Fig. 3B–D). We find that these factors frequently shape the characteristic *Rhododendron* species of specific regions (or provinces) (Supplementary Note S3).

Based on the recently published genome of *Rhododendron*, we further reconstructed the phylogenetic tree among *Rhododendron* species, incorporating more high-quality genomes (Fig. 3E). In the traditional classification of *Rhododendron*, there are primarily five subgenera: *Hymenanthes*, *Rhododendron*, *Tsutsusi*, *Azaleastrum*, and *Pentanthera* [1, 2]. Among these, the subgenus *Hymenanthes* exhibits the greatest species diversity [1]. In the phylogenetic tree reconstructed, the evolutionary relationships and classification of *Rhododendron* are generally consistent with previous studies [2, 22]. However, it is noteworthy that the species *R. bailiense* is classified under the subgenus *Rhododendron* in this study, which differs from the previous morphological classification placing it in the subgenus *Hymenanthes* [13]. Our previous research has indicated that *R. bailiense* appears to be evolutionarily distinct from the traditional subgenus *Hymenanthes* species [2]. However, due to limited genomic resources at that time, we were unable to definitively clarify which subgenus *R. bailiense* belongs to. Based on the recently published genomes of *Rhododendron concinnum* and *R. liliiflorum* [21], we have established that *R. bailiense* is classified under the subgenus *Rhododendron*. This provides new insights into understanding the systematic evolutionary origins of *R. bailiense*.

Previous studies have suggested that these characteristics are closely related to the species formation and phylogenetic classification of rhododendrons [5]. Overall, the subgenus *Hymenanthes* of rhododendrons grows in low nitrogen-phosphorus soils and has lower specific leaf area (SLA) values, while the subgenus *Pentanthera* thrives in high nitrogen-phosphorus soils and possesses higher SLA values [5]. The subgenera *Tsutsusi* and *Azaleastrum* are positioned at an intermediate level. Due to these differences in soil nutrient characteristics, they are often associated with plant responses to drought and nutrient deficiency stresses, which are further linked to Ca^2+^ signal-mediated stress response processes [6, 14], and subsequently related to the Ca^2+^ transport processes mediated by CAPs. We conclude that the characteristics of different *Rhododendron* CAPs are associated with their habitats. Interestingly, we found that the overall copy number of CAPs in *Hymenanthes* and the *Rhododendron* subgenus compared to the *Tsutsusi*, *Azaleastrum*, and *Pentanthera* subgenera in low-altitude areas is greater (Figs 2B and 3B, D, E). This trend correlates with the nutritional characteristics of the soils in which different *Rhododendron* species are found. Specifically, rhododendrons in low-nutrient soil areas have a higher copy number of CAPs, while those in high-nutrient soil areas have a lower copy number. It is noteworthy that the three *Rhododendron* species distributed in the Guizhou BRNR, namely, *R. irroratum*, *R. bailiense*, and *R. delavayi*, possess the highest copy number of CAPs. This indicates that the unique karst geographical environment of Guizhou has shaped the genotype of the three rhododendrons (for more cases, see Supplementary Note S4). The karst topography in Guizhou is typically rich in Ca^2+^, which requires Ca^2+^ transport proteins for transport and processing to avoid high calcium toxicity [14, 15]. This precisely reflects the reason for the high copy number of CAPs in the Guizhou *Rhododendron*. Compared to herbaceous model plants like *Arabidopsis* and rice, which possess only a limited number of CAP copies, the massive expansion of CAPs in the *Rhododendron* species of Guizhou highlights a unique evolutionary trajectory. This pronounced expansion in karst-adapted species (e.g. *R. irroratum* and *R. bailiense*) mirrors the adaptive genomic strategies observed in other extremophiles [4, 35], underscoring the critical evolutionary role of CAPs (Ca^2+^ pumps) in mitigating calcium toxicity in specialized high calcium habitats. In summary, the derived information above suggests a close relationship between the habitat in which rhododendrons are located and the number of CAP copies.

### The evolutionary history and natural selection of CAPs in rhododendrons

**Figure 4:**
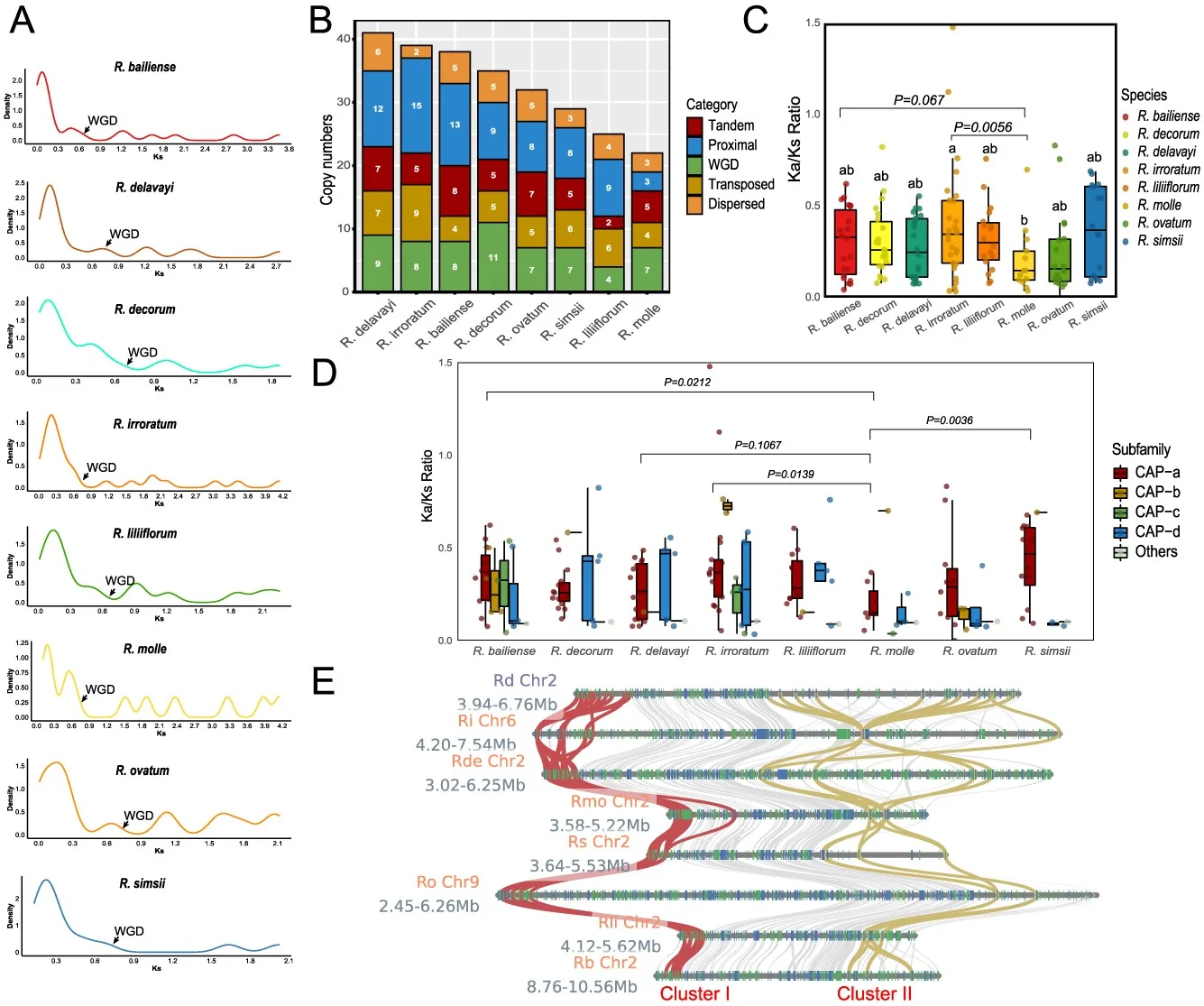
The duplication characteristics of CAPs and natural selection. (A) The synonymous mutation rate (Ks) of CAPs gene pairs in various *Rhododendron* species. A lower Ks value indicates that the duplication event occurred more recently. (B) Comparison of the number of different duplication types of CAPs gene pairs among various *Rhododendron* species. (C) Box plots showing the comparison of nonsynonymous substitution levels (Ka)/Ks among CAPs gene pairs in different *Rhododendron* species. Letters a and b indicate significant differences in data (*P* < 0.05), using Tukey’s test. The ab category represents transitional data with no significant difference from either a or b. (D) Box plots comparing the distribution of Ka/Ks among different subfamilies of different species. *P* values for several groups with significant differences are annotated in the figure, with significance assessed using the *t* test. (E) The synteny relationships of two CAPs gene clusters on *Rhododendron* Chr2 (*R. irroratum*, Chr6; *R. ovatum*, Chr9).

We aim to explore the origin patterns and evolutionary history of CAPs in *Rhododendron*. The Ks distribution of CAPs across all *Rhododendron* species was first analyzed (Fig. 4A). The results indicate that the peak of Ks for all *Rhododendron* CAPs occurs at lower positions, while there is only a slight increase at the whole-genome duplication (WGD) positions, suggesting that the primary source of CAPs is recent high-frequency duplications. Integrating the phylogenetic tree of *Rhododendron* (Fig. 3E), it can be inferred that most CAPs originated after the emergence of *Rhododendron* species. Furthermore, subgenus *Hymenanthes*, including species such as *R. irroratum* and *R. delavayi*, exhibits higher peak density values compared to *R. molle*, indicating that CAP duplication events in *Hymenanthes* have occurred more frequently in recent times. Additionally, in terms of the overall Ks distribution of genes, *Rhododendron* plants predominantly show Ks values concentrated at lower levels, indicating that they have largely experienced frequent gene duplication events recently (Supplementary Fig. S5). Therefore, the frequent duplication of CAPs is part of these recent gene duplication events. Such high-frequency gene duplications, often caused by tandem duplications (TD), are quite common in wild plants [30, 36], suggesting an evolutionary strategy adopted by *Rhododendron* plants to adapt to their environment. Interestingly, through a summary of the duplication types, we found that TD and proximal duplications (PD) predominantly governed the duplication of CAPs in rhododendrons (Fig. 4B). This feature is most pronounced in the three *Rhododendron* species sampled from BRNR: *R. bailiense*, *R. delavayi*, and *R. irroratum*.

Through the analysis of the Ka/Ks ratio, we found that the majority of CAPs have a Ka/Ks ratio of less than 0.5. Considering the stability of CAPs structure (Supplementary Fig. S1), this suggests that most CAPs have undergone purifying selection. From a species perspective, *R. simsii*, *R. bailiense*, and *R. irroratum* have experienced relatively weak purifying selection, while *R. molle* has undergone the strongest purifying selection (Fig. 4C). This may stem from the fact that *R. simsii*, *R. bailiense*, and *R. irroratum* have experienced changes in elevation ecological niches during evolution, leading to a moderate variation requirement for CAP genes. In contrast, *R. molle* is predominantly distributed in low-altitude areas, which may impose strong purifying selection on CAPs as its dominant evolutionary pressure. A more detailed analysis revealed that the Ka/Ks of CAP-a in *R. bailiense*, *R. irroratum*, and *R. simsii* is significantly higher than that of *R. molle* (Fig. 4D). This is the main factor for the difference in selective pressure between *R. molle* and other rhododendrons. From the perspective of subfamilies, CAP-a, CAP-b, and CAP-d exhibit members that have undergone relatively strong positive selection, with CAP-a showing a more uniform Ka/Ks distribution (Supplementary Fig. S6). This indicates the presence of CAP-a copies under varying degrees of selection. In contrast, CAP-e and CAP-f are subjected to the strongest purifying selection (Supplementary Fig. S6). This strong conservation may reveal the reason that CAP-e and CAP-f are positioned early in the phylogenetic tree and have been retained for a long time (Fig. 1A). By integrating the chromosomal location information of all *Rhododendron* CAPs, we found that their CAPs are concentrated in a large gene cluster approximately 2 Mb in length on Chr2, which is divided into two sections (Cluster I + II) (Fig. 4E). This explains the primary sources of TD and PD for CAPs. These two gene clusters are conserved across all *Rhododendron* species, with a gap between them, and importantly, the genes in both gene clusters belong to the CAP-a subfamily.

Our previous results indicate that different species of *Rhododendron* possess varying copy numbers of CAPs, but the mechanisms behind the generation of these CAPs remain a question. While there has been extensive research on the functional aspects of CAPs in *Arabidopsis* [6], the evolutionary patterns and generation mechanisms of CAPs in wild, nonmodel plants have been rarely explored. Previously, we discovered widely duplicated CAPs in *R. bailiense* [2], but there is still insufficient investigation in other *Rhododendron* species. Here, we found that recent widespread TD and PD events in *Rhododendron* species frequently occur, leading to a generally high copy number of CAPs in *Rhododendron* species such as *R. delavayi* and *R. irroratum*, both of which are found in Guizhou. We found that CAPs generally underwent purifying selection, with *R. molle* experiencing the highest degree of purifying selection. This significant proportion of purifying selection reflects the relatively stable functional characteristics of CAPs throughout their evolutionary history. Furthermore, these CAPs belong to the CAP-a subfamily and are clustered on Chr2. The frequent occurrences of TD and PD events are also common in other genes of *Rhododendron*. For instance, the terpene synthase genes (TPSs) in *Rhododendron ovatum* enhance its low-altitude adaptability through TD and PD [36]. In *R. bailiense*, the chalcone synthase (CHS) promotes flavonoid synthesis through extensive duplication [25]. These studies indicate that *Rhododendron* often amplifies stress-related genes through TD and PD. Overall, the results in this section elucidate the frequent duplication patterns of CAPs at the pan-genome level and the key CAP clusters in *Rhododendron*.

### Analysis of comprehensive expression patterns of CAPs in rhododendrons

**Figure 5:**
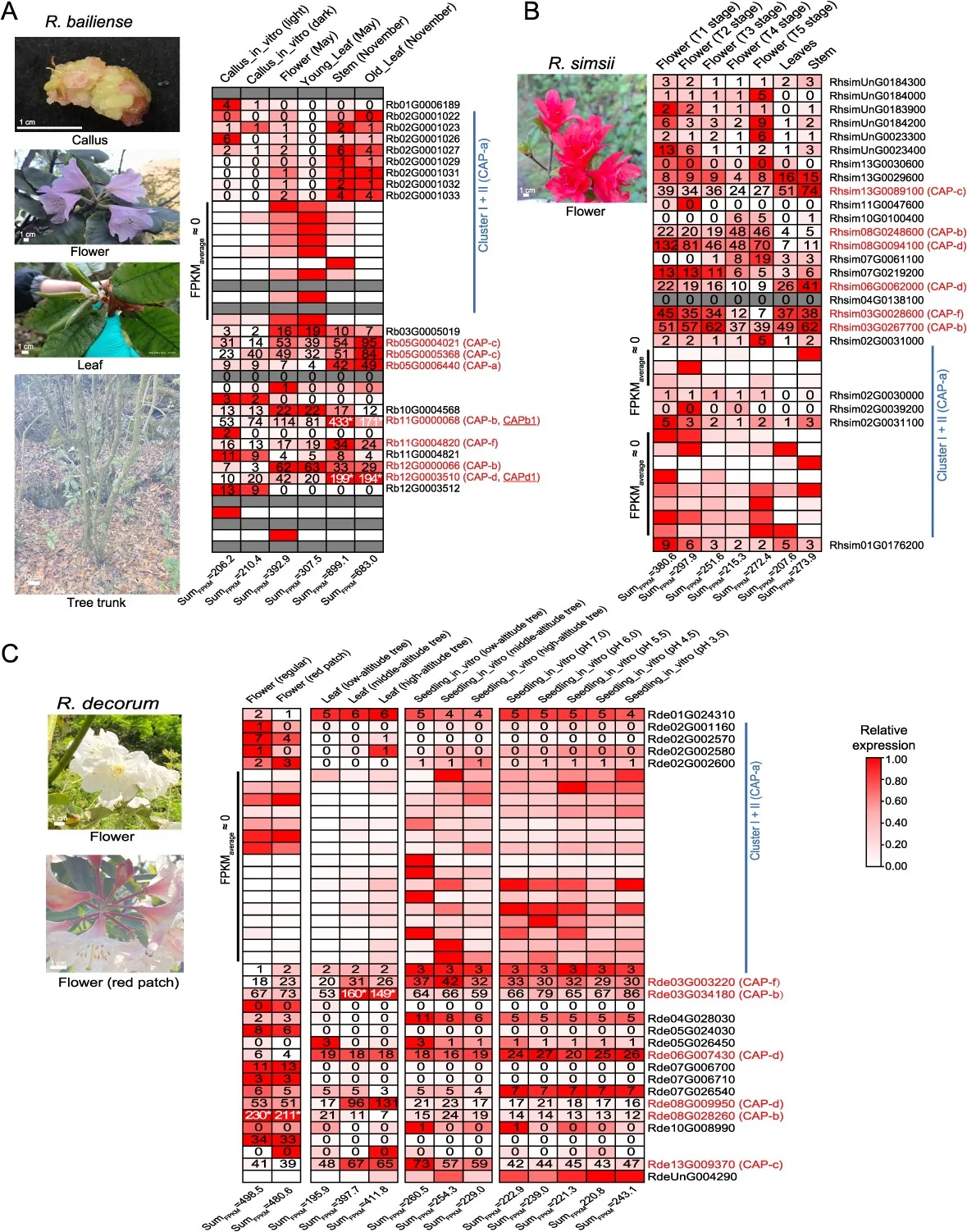
Expression patterns of CAPs under different conditions. (A–C) Phenotypes of *R. bailiense*, *R. simsii*, and *R. decorum*, along with the expression patterns of CAPs in various tissues. The gray squares indicate nonexpressing genes. Genes with particularly high expression levels are marked with an asterisk. The numbers within the squares represent the FPKM values of gene expression. CAP genes with expression levels consistently low are not assigned FPKM values (<1). For members CAP-b, CAP-c, CAP-d, and CAP-f, at least one member typically shows higher expression (marked with red names). Most CAP-a genes (primarily belonging to Cluster I + II) are at low expression levels. The total FPKM values of CAPs for each condition are indicated below the heatmap, revealing the overall level of CAPs expression in that condition. The heatmap presentation utilizes a method of row-wise normalization.

In this section, we utilized newly sequenced and publicly available transcriptomes from various species of rhododendrons to explore the expression of CAPs under wild conditions and *in vitro* cultured laboratory conditions. Various plant habitat conditions were also integrated to comprehensively understand the impact of different conditions on CAPs expression. The primary focus was directed to *R. bailiense* in the initial phase of analysis. Through transcriptome analysis of wild materials (flowers, young leaves, stems, old leaves) and *in vitro* cultured materials (callus under light treatment and dark culture), we found that, except for CAP-e, at least one to two members of the CAP-a to CAP-f family were in a relatively high expression state (Fig. 5A). This suggests a ‘baseline’ expression pattern of these family members. It is noteworthy that the CAP-a subfamily, comprising members of Cluster I and II, exhibits very low expression levels, with many members being nearly silent. Only in stems and old leaves do some CAP-a members exhibit weak expression. Therefore, CAP-a members possess a unique expression pattern, resembling a ‘storage repository’, where genes are expressed only under specific conditions. On a broader scale, the overall expression levels of CAPs in wild materials are significantly higher than those in *in vitro* cultured callus, as evidenced by the differences in the total FPKM values (Fig. 5A). Additionally, it is important to highlight that *Rb11G0000068* (CAPb1) and *Rb12G0003510* (CAPd1) belong to the CAP-b and CAP-d subfamilies, respectively, both of which are highly expressed in stems and old leaves (collected in November). Specifically, CAPb1 has an FPKM value of 433 in stems, while the total FPKM of all CAPs in stems is 899.1, which is the highest among all cases involving rhododendrons in this study. These results suggest that CAPb1 and CAPd1 may play crucial roles in the response to stress and aging processes in stems and old leaves.

**Figure 6:**
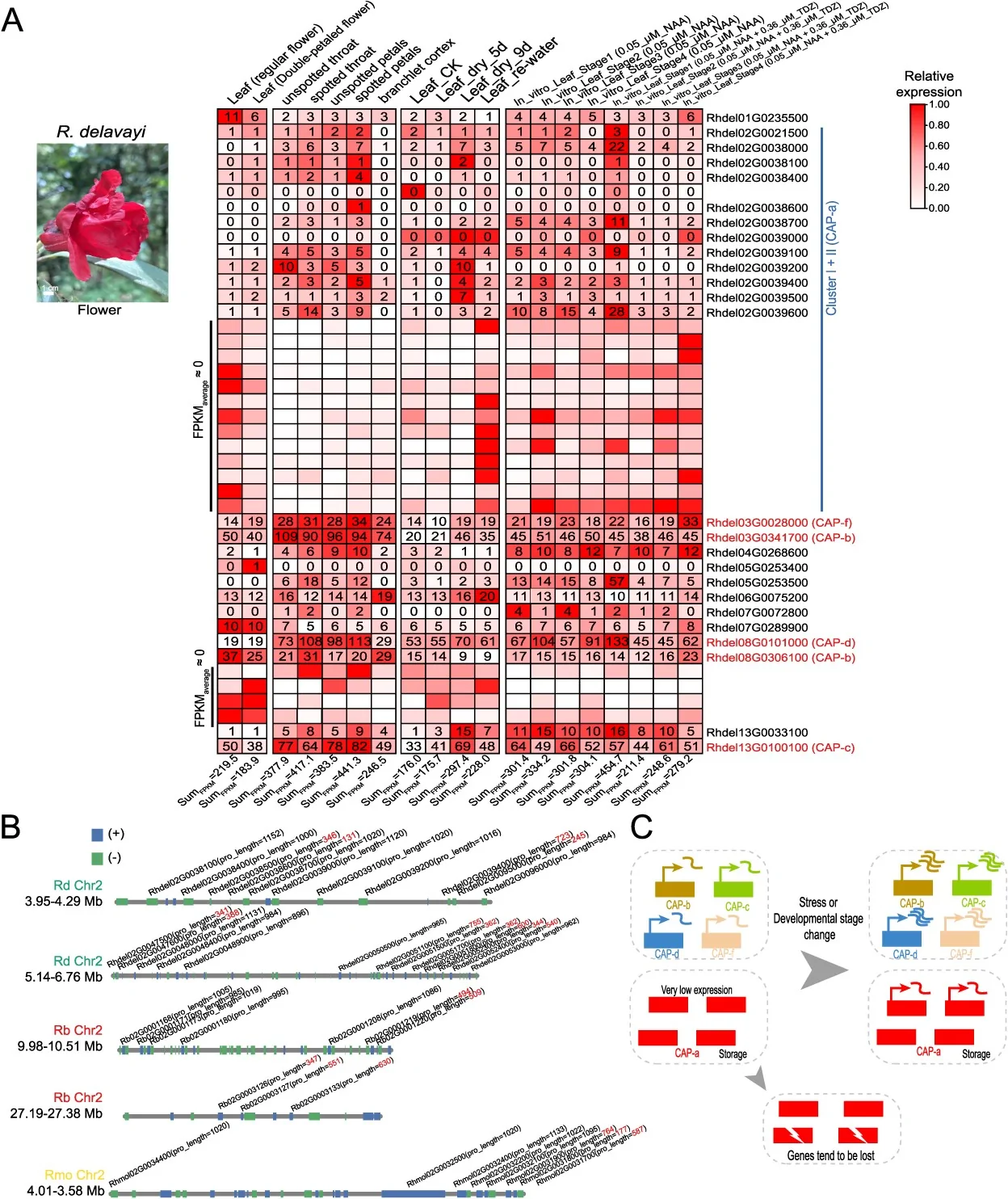
Expression profiles of CAPs in *R. delavayi* and an overview of CAPs expression patterns and presence across *Rhododendron* species. (A) Phenotype of *R. delavayi* and the expression patterns of CAPs in various tissues and conditions. The numbers within the squares represent the FPKM values of gene expression. The heatmap presentation utilizes a method of row-wise normalization. (B) Distribution and protein length information of CAP-a subfamily genes on Chr2 in *R. delavayi*, *R. bailiense*, and *R. molle*. The gene names are annotated with the protein lengths (longest transcript). Members with significantly shortened lengths (i.e. premature termination) are highlighted in red. (C) Schematic summary of the expression and presence patterns of various CAP subfamilies. CAP-b to CAP-d and CAP-f typically have at least one member expressed at low levels under normal conditions; under specific stress or developmental conditions, some members were induced to express at high levels. For example, the expression level of CAP-b in high-altitude *R. decorum* is three times higher than in low-altitude environments. CAP-a is generally in a state of extremely low expression or not expressed at all under normal conditions, but can be induced to express at low levels under specific conditions.

In *R. simsii*, we found that CAP-d is highly expressed during the early stages of flower development (Fig. 5B; Supplementary Note S5). In *R. decorum*, an interesting phenomenon is the high expression of specific members of CAP-b and CAP-d in high-altitude environments (Fig. 5C). Similarly, in *R. delavayi*, CAP-b and CAP-d also exhibited high expression in floral organs and under TDZ (thidiazuron) treatment (Fig. 6A; Supplementary Note S5). These results, along with further transcriptomic data analysis, collectively indicate that the expression of CAPs is induced and regulated by various environmental factors and developmental stages (Supplementary Figs S7 and S8; Supplementary Note S5). In all the cases of expression, the CAP-b and CAP-d subfamilies encompass the largest number of highly expressed members, as well as the gene with the highest expression level. This phenomenon is also observed in *Arabidopsis* [11].

The traditional laboratory stress theories need a transitional framework when applied to the survival status of plants in their natural habitats, as the field environment is significantly more complex than the conditions imposed in laboratory settings [34]. Here, we used rhododendrons as the research material to investigate the expression patterns of various CAPs under different laboratory and wild conditions, as well as in various organs. The species of *Rhododendron* exhibit the following characteristics in their evolutionary process: they enhance their environmental adaptability by frequently duplicating adaptive genes; they further improve environmental adaptability through the high expression of highly copied genes [4]. Therefore, specific highly expressed members within the high-copy gene family may play a crucial role in supporting the expression levels of a particular family. Our results highlighted the high expression of specific CAP-b and CAP-d genes in the stems and old leaves of *R. bailiense*, which, combined with previous studies [6, 31, 37], strongly suggest their role in stress response functions in *R. bailiense* that has grown long-term in karst environments. Notably, the specific homologous genes of CAP-b (i.e. *Rb11G0000068*, *Rhdel03G0341700*, *Rde03G034180*, *Rhsim03G0267700*) and CAP-d (i.e. *Rb12G0003510*, *Rhdel08G0101000*, *Rde08G009950*, *Rhsim08G0094100*) in nearly every *Rhododendron* species exhibited the highest expression levels and were induced to the greatest extent under certain conditions (e.g. stress and flower development) (Figs 5 and 6). Taken together, we identified key candidate genes (e.g. CAPb1 and CAPd1 in *R. bailiense*) by integrating phylogenetic conservation and specific stress-induced expression profiles. This integrative screening strategy suggests that the selected genes likely play vital roles in high calcium adaptation. CAP-b is primarily located on the plasma membrane, while CAP-d is mainly found on the endoplasmic reticulum. Research on homologous genes in *Arabidopsis* indicates their significant roles in pollen tube, root, and leaf development, as well as in stress resistance [6, 10, 11], supporting our viewpoint. Additionally, we discovered the uniqueness of the CAP-a subfamily in *Rhododendron*, where its members are extensively duplicated but remain in a low expression state, with only a subset being induced under specific conditions. In addition to interspecies variations, we examined the copy number variation of CAP-a within the *R. delavayi* population. Our findings revealed that the variation of CAP-a on Chr2 is highly frequent among different individuals (Supplementary Fig. S9), indicating that this locus is a hotspot for variation. Many of these CAP-a proteins have undergone premature termination (Fig. 6B). CAP-a generally exhibits low Ks and recent duplication characteristics. Combined with the ‘balanced expression’ theory among homologous genes (i.e. low-expressed copies are prone to premature termination and loss) [38], it indicates that CAP-a is in a rapid cycle of birth and extinction during its evolutionary process. We define this expression pattern as a ‘storage expression mode’, and the related mechanistic model was summarized in Fig. 6C. Under normal conditions, *Rhododendron* plants suppress the expression of CAP-a members to avoid energy wastage and metabolic disorder. However, when subjected to stress, they release the ‘storage’ gene pool for expression to combat adverse conditions. This is evidently a strategy for plants to balance ‘growth’ and ‘stress response’, which partially addresses the question posed by Taishi Umezawa in 2022 regarding how plants balance growth and abiotic stress responses [34]. Similar expression patterns have also been reported in other plant genes [38]. Overall, in this section, we systematically summarize the expression patterns of CAPs under different conditions at the pan-genomic level in *Rhododendron* and provide new insights into their unique expression patterns.

### Co-expression network of CAPs and associated gene analysis

**Figure 7:**
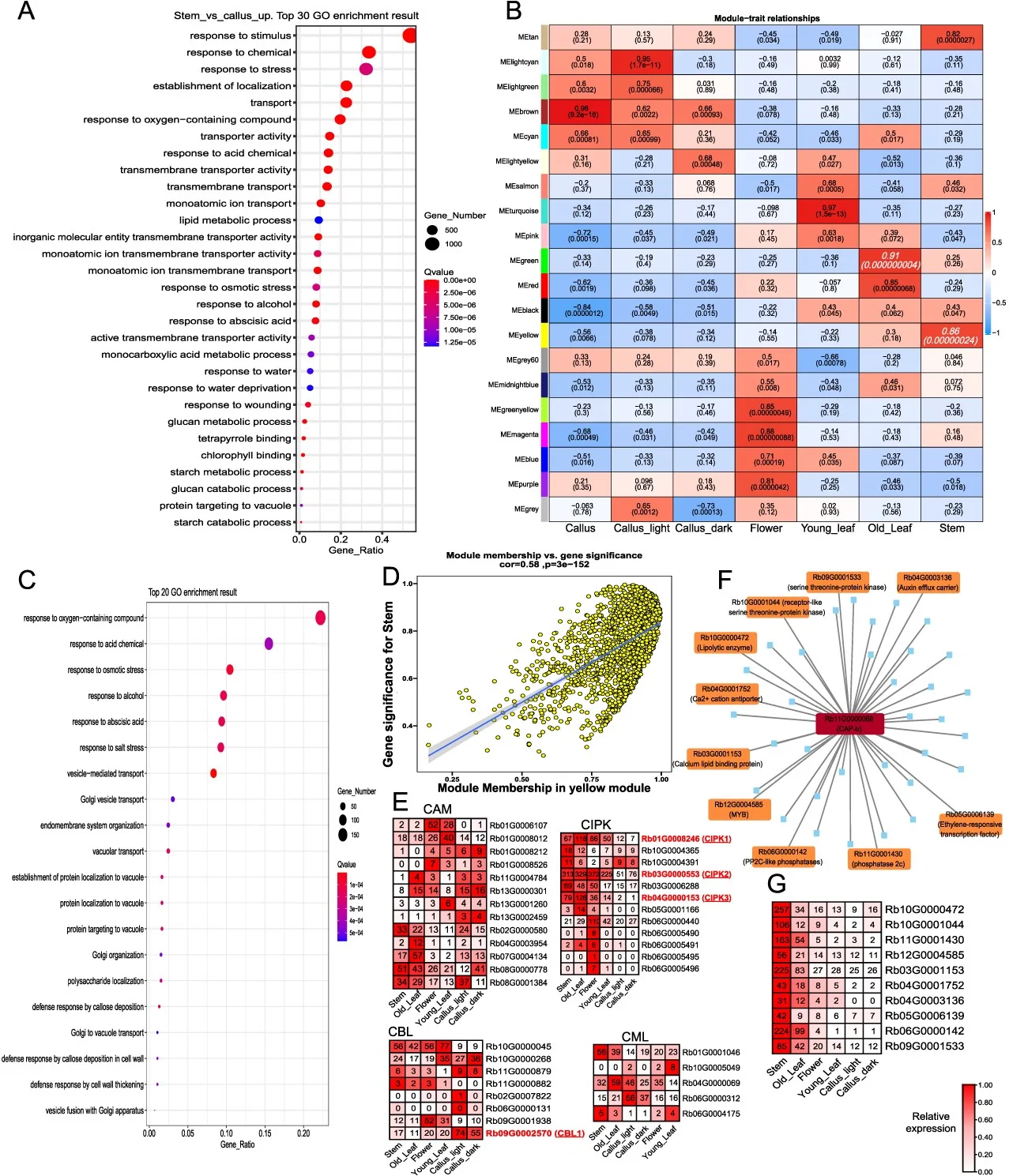
Co-expression network of CAP-b. (A) GO enrichment analysis reveals functional information of upregulated genes in the stems of *R. bailiense* compared to callus. (B) The heatmap displays the correlation coefficients between modules and different tissue samples of *R. bailiense*. The correlation coefficients between modules and samples are represented by the colors of the cells and the text within them (the upper part shows the Pearson correlation coefficients, while the lower part shows the *P* values). The association value of MEyellow (containing CAPb1) with stem, and the association value of MEgreen (containing CAPd1) with old leaf are relatively high, marking them as key research targets, highlighted in italic white font. (C) GO functional enrichment analysis of genes in MEyellow. (D) The relationship between module membership and gene significance for genes in MEyellow. (E) The heatmap displays the expression patterns of calmodulin (CAM), calcineurin B-like protein (CBL), CBL-interacting protein kinases (CIPK), and calmodulin-like protein (CML) in different tissues or under various conditions, which have potential interaction relationships with CAP-b in the context of *R. bailiense*. The values within the squares represent the FPKM values of gene expression. (F) A co-expression network closely associated with *Rb11G0000068*. Genes that were selected for their potential significant functions and critical connections are represented by orange squares, with gene names and loci annotated. (G) The expression patterns of the identified potential key co-expressed genes.

In order to further understand the associated genes and regulatory genes expressed by CAPs, we conducted a comprehensive analysis. Primary focus was directed to *R. bailiense*, with comparative analysis of differential gene expression patterns between wild-state stems and *in vitro* cultured callus being conducted. Through enrichment analysis of differentially expressed genes, we found that the genes highly expressed in the stems were concentrated in categories such as ‘response to stimulus and stress’ (Fig. 7A), whereas the highly expressed genes in the callus were primarily associated with ‘nucleic acid metabolic process’ (Supplementary Fig. S10). This reveals a fundamental difference in gene expression patterns between wild stems and *in vitro* cultured callus, with numerous stress response genes being highly expressed in the stems.

To explore genes with expression associations with CAPs, we further performed weighted gene co-expression network analysis (WGCNA) in *R. bailiense*. The results integrated gene expression into several modules (Supplementary Fig. S11A), and notably, the MEyellow module, which had the highest correlation with the stems, included the key CAP-b gene *Rb11G0000068* (Fig. 7B). We conducted an enrichment analysis of the genes within the MEyellow module and found that their functions are concentrated on stress response functions related to ‘response to oxygen-containing compound’ and ‘Golgi vesicle transport’ (Fig. 7C), suggesting that the stems of *R. bailiense* are in a state of continuous stress. The genes in the MEyellow module show a close relationship with the stems (Fig. 7D) and are predominantly expressed at high levels in the stems (Supplementary Fig. S12A). To further investigate the co-expression relationships of *Rb11G0000068* (CAP-b) with other genes, we conducted an exploratory analysis (Fig. 7F). Through gene filtering, several interesting candidate genes were identified, such as ‘serine threonine-protein kinase’, ‘Ca^2+^ cation antiporter’, ‘calcium lipid binding protein’, and ‘MYB’, among others (Fig. 7F; Supplementary Data Set S2). The selected co-expressed genes exhibit high expression levels in the stems of the *R. bailiense* (Fig. 7G), suggesting their involvement in the cooperative physiological processes related to CAP-b. Calmodulin (CAM), calmodulin-like protein (CML), calcineurin B-like protein (CBL), and CBL-interacting protein kinases (CIPK) are other proteins that have been reported to interact with autoinhibited CAPs through their N-terminal regions [7] (Supplementary Data Set S3). Some members among them, such as CIPK2 and CIPK3, exhibit high expression levels (Fig. 7E). Highly expressed genes may be potential interacting members of activation pathways. We further investigated the co-expression gene types of CAP-b and CAP-d in more rhododendrons and found that environmental response genes predominated. For more details, please refer to Supplementary Note S6 and Supplementary Figs S12–S19.

In this section, we conducted a comprehensive analysis of the co-expression network of CAPs in various rhododendrons. Previous research has indicated that CAPs interact with genes such as CIPK and CBL [7], yet studies on their co-expressed genes have been scarce. In our findings, we utilized transcriptomic resources from rhododendrons and discovered that the expression of CAPs is often accompanied by the high expression of stress response-related genes. This result extends the functional information of CAPs established in previous studies [10]. The expression of CAPs is typically associated with an increase in cytosolic Ca^2+^ concentration, which is usually linked to enhanced defensive responses. However, excessive defensive responses can adversely affect plants, potentially leading to death. Therefore, plants need to pump out excess calcium ions via CAPs to ensure normal physiological metabolism. Our results provide a comprehensive expression pattern of genes within the plant during high expression of CAPs from a gene expression perspective, which has been less elucidated in the past. We found that the expression of CAP-b and CAP-d is typically accompanied by serine threonine-protein kinases, which may be involved in stress signal transduction. Additionally, in CAP-d, the active E3 ubiquitination process indicates that the expression of CAP-d is associated with the degradation of stress-related proteins, which complements previous studies [10]. This is because the high expression of CAPs is considered a process that mitigates stress responses. The high expression of CAP-d is also associated with the activity of vacuolar protein sorting-associated proteins and the nucleotide-binding leucine-rich repeat (NB-LRR) family (Supplementary Fig. S16D), suggesting that vacuolar activity is linked to immune responses during this process. Furthermore, our findings in this section indicate that MYB and ethylene-responsive transcription factor (ERF) may be direct regulatory transcription factors of CAPs (Supplementary Fig. S16F). In summary, this section elucidates the co-expression network of CAPs in rhododendrons, providing names and locations of co-expressed genes. This section also enhances our understanding of the molecular networks and physiological activities related to CAPs, and lays the groundwork for more specific gene studies.

### Functional analysis of two key CAP members

The structural characteristics of membrane proteins are fundamental to their molecular functions. To further understand the structural variations of CAPs from an evolutionary perspective, CAPs were selected from *Rhododendron* species, including *R. bailiense*, *R. delavayi*, and *R. simsii*, as representatives to study the structural features of different subfamilies of CAPs. For the CAPs clustered by sequence alignment from CAP-a to CAP-f, upon predicting their structures, we found that the structural differences among the subfamilies are minimal (Supplementary Fig. S20). Taking *Rb02G0001032* as an example, the typical autoinhibited CAPs domain is divided into the AD, A-domain, N-domain, P-domain, and transmembrane domains (TMs) (Supplementary Fig. S20B, right inset). All CAPs generally adhere to this structural basis, with the exception of the ER-type CAPs (belonging to the CAP-c subfamily), which lack the AD domain and exhibit slight differences; simultaneously, CAP-c has the least shared motifs, indicating the sequence uniqueness of these ER-type CAPs (Supplementary Fig. S20A).

**Figure 8:**
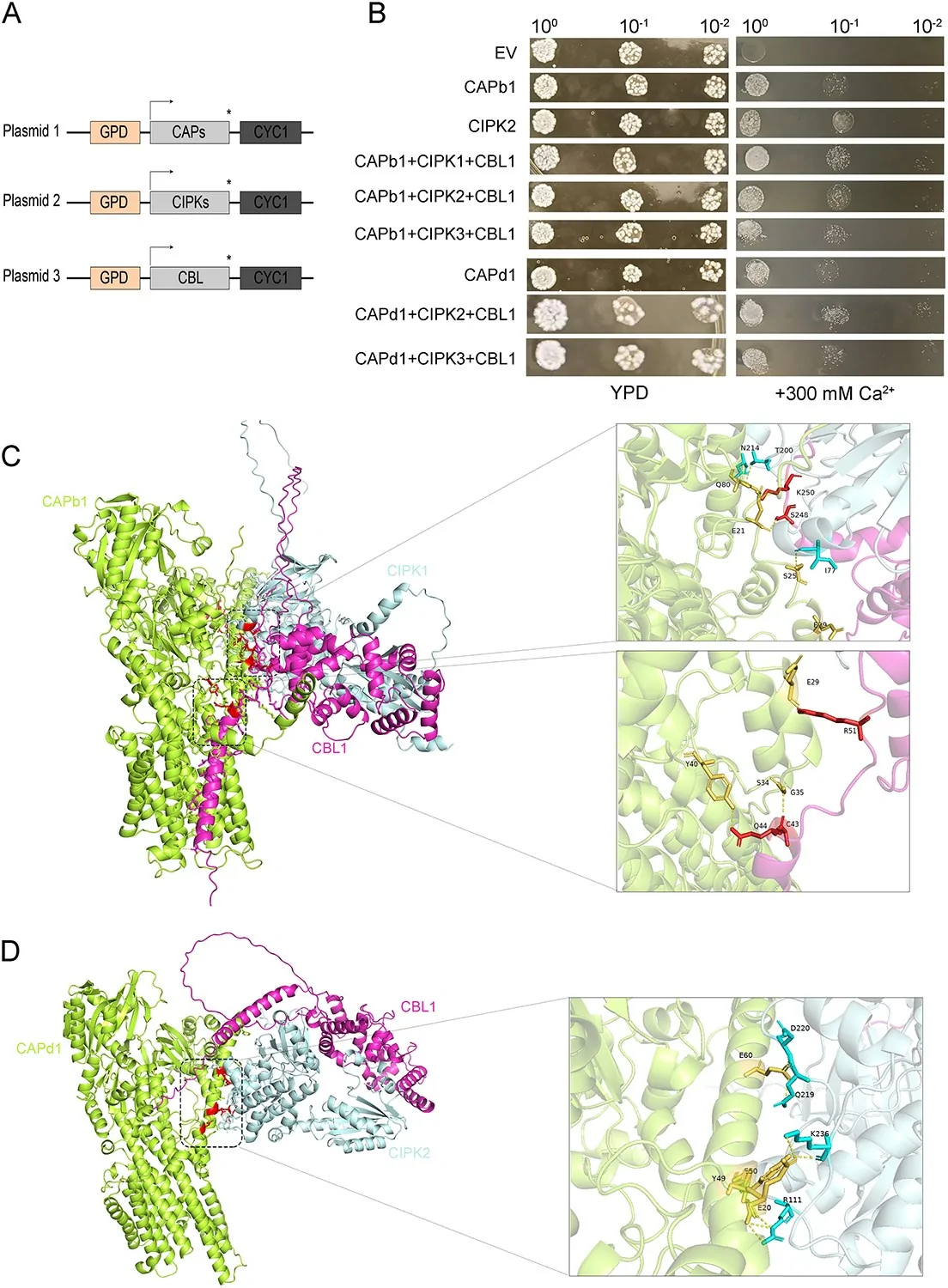
Functional validation of CAPb and CAPd and their synergistic effects with CIPK-CBL. (A) Recombinant plasmids used for co-expressing proteins in yeast. GPD is a strong promoter in yeast, and CYC1 is the terminator. (B) Co-expression of CAPb1/CAPd1 with CIPKs and CBL1 in the yeast strain BY4741, grown on YPD and YPD + 300 mM Ca^2+^ media. The figure shows representative images from two independent experiments. EV, empty vector. (C and D) Interaction structural models of CAPb1-CIPK1-CBL1 and CAPd1-CIPK2-CBL1 predicted based on AlphaFold3. (Inset figure) An enlarged view of amino acid interactions (marked) between proteins. Yellow dashed lines indicate hydrogen bonds.

Wang *et al.* established a yeast system for the functional study of calcium channel proteins, which can simultaneously identify the effects of calcium channel proteins and ligand proteins in response to high calcium levels, and it shows good reproducibility in plants [15]. To investigate the functions of the main members of CAPs, we appropriately adjusted this system and employed a strategy of transforming three plasmids into functional yeast (BY4741) at once, to study the synergistic response of CAPs, CIPKs, and CBLs to high calcium stress (Fig. 8A). We focused on the highly expressed and relatively intact structures of CIPKs and CBLs (Fig. 7E). Interestingly, when the nine transformed yeast strains were cultured on conventional yeast-extract peptone dextrose (YPD) medium, they all grew normally (Fig. 8B). However, when transformed with an empty vector (EV), the yeast could not grow normally under high calcium stress (300 mM). Notably, when the transformed targets were CAPb1, CIPK2, or CAPd1, the yeast could significantly recover growth (Fig. 8B), indicating that these targets alleviated the high calcium stress experienced by the yeast. The reason that the transformation into CIPK2 can restore growth may be attributed to the presence of a small number of CAP gene copies in yeast. Furthermore, the co-expression of the CIPK1-CBL1 module with CAPb1 resulted in a markedly enhanced colony density and survival rate compared to the expression of CAPb1 alone (Fig. 8B), confirming their strong synergistic effect in alleviating calcium toxicity. Similarly, CIPK2-CBL1 notably enhanced the function of CAPd1 in resisting high calcium stress, outperforming other combinations of CIPKs and CBL1 (Fig. 8B). This indicates that the CIPK-CBL module differentially regulates CAP activity, with specific CIPK/CBL combinations identified as key determinants of efficiency, and can significantly improve the alleviation effect against high calcium toxicity. To understand the interaction characteristics between CAPs and CIPKs-CBLs at the structural level, AlphaFold3 was utilized to predict the structural model of CAPs-CIPKs-CBLs. For CAPb1, the complex formed by CIPK1 and CBL1 binds to the AD region of CAPb1, and CIPK1-CBL1 may have a dual binding affinity with CAPb1 (Fig. 8C). In the case of CAPd1, structural predictions indicated that CBL1 did not appear in the proximal region of CAPd1, primarily involving the interaction between CIPK2 and CAPd1 (Fig. 8D). This suggests two binding modes between CIPKs-CBLs and CAPs. A possible reason is that CAP-b1 possesses a longer AD, which may allow it to interact simultaneously with CIPK-CBL. This warrants further investigation in the future.

The functional research on CAPs has emerged as a hot topic in recent years [10, 11]. Although CAPs in *Arabidopsis* are known to regulate calcium balance, promote growth, and facilitate pollen maturation [10, 11], studies on their role in woody plants are scarce, particularly regarding the activation of kinase proteins. Wang *et al.* previously demonstrated that CIPKs-CBLs activate the activity of vacuolar Ca^2+^/H^+^ exchangers (CAXs) through interaction, alleviating high calcium stress [15]. For CAXs or CAPs that possess AD, the phosphorylation of the AD region is a key determinant of transport activity [15]. Specific CIPKs-CBLs can more effectively phosphorylate the AD phosphorylation of certain CAPs, leading to an increase in activity. Therefore, the phosphorylation properties of the protein itself determine the transport activity, a principle that is consistently observed in both yeast and plants, demonstrating good reproducibility. Thus, the yeast system developed by Wang *et al.* can effectively reveal the efficacy of calcium transport proteins in plants and their interaction levels with partner proteins [15]. Here, we utilized the well-established yeast system to demonstrate that both CAPb1 and CAPd1 in *R. bailiense* have the capability to alleviate high calcium stress [15]. Given the high homology of CAPs in rhododendrons, it can be inferred that the CAP proteins in other *Rhododendron* species also possess the ability to mitigate high calcium stress. Currently, we are working on developing a genetic transformation system related to rhododendrons and have made preliminary progress [25]. We hope to utilize CAPs in the future to develop high calcium tolerant rhododendron plants. Furthermore, specific members of the CIPK-CBL family enhance the high calcium alleviation capacity of CAPb and CAPd, indicating that they can strengthen the activity of CAPs and further improve the adaptability to high calcium stress [15]. The karst landscape prevalent in southwestern China is rich in Ca^2+^ [14]. These findings underscore the significant role of CAPs and the CAPs-CIPKs-CBLs module in alleviating high calcium stress, elucidating part of the reasons and strategies for the diverse species of rhododendrons to thrive in the high calcium environment of the southwestern Chinese karst region.

## Conclusion

In conclusion, our results indicate that CAPs emerged and diversified early in plant evolution, followed by significant amplification in certain plant lineages during subsequent evolutionary processes. We also systematically summarize the copy number variations of CAPs, their evolutionary patterns, and their close relationship with the living environment at the pan-genome level among different species of *Rhododendron*. Our results indicate that the CAP-b to CAP-d subfamilies in *Rhododendron* are easily induced by conditions such as karst and high-altitude environments. Furthermore, the CAP-a subfamily exhibits a ‘storage’ type of expression strategy, which is significantly different from other CAP subfamilies in terms of evolutionary and expression attributes. We have summarized the co-expression networks associated with CAP-b and CAP-d, along with key related genes and loci, where stress-related genes dominate. Yeast validation experiments have demonstrated that CAPb1 and CAPd1 possess significant abilities to alleviate high calcium stress. Moreover, the CAPb1-CIPK1-CBL1 and CAPd1-CIPK2-CBL1 modules can further enhance the function of alleviating high calcium stress. However, current research is limited to the functions of only a few CAP members. Future studies may focus on utilizing CAPs and stable genetic transformation techniques to construct high calcium-tolerant rhododendron or other plant varieties, as well as how CAPs collaborate with CAXs to regulate plant adaptability and immunity. The findings of this study provide new insights into the evolution, characteristic expression, and function of plant CAPs, as well as new understanding of how *Rhododendron* adapts to high calcium environments. This theoretical knowledge may be applied to the stress resistance screening/breeding of *Rhododendron* and other excellent forest tree germplasm, as well as in the management of high calcium karst desertification environments.

## Materials and methods

### Plant materials and transcriptome resources

We collected four species of *Rhododendron* resources from the Guizhou Baili Rhododendron Nature Reserve (BRNR), which is characterized by a subtropical plateau monsoon humid climate. These include *R. bailiense*, *R. delavayi*, *R. decorum*, and *Rhododendron agastum*. Among them, *R. bailiense* grows in a shrub forest at the top of a karst landform peak (27°14′24″N; 105°52′42″E), where the soil is thin and the tree roots are nearly embedded in the rocks of the karst mountain. In May 2023, we collected young leaves and flowers from a *R. bailiense* tree, and in November 2023, we collected old leaves, stems, and seeds from the same tree. For the collected seeds, after disinfection with sodium hypochlorite and five washes with distilled water, they were sown on woody plant medium (WPM) supplemented with 1.0 mg·l^−1^ kinetin (KT) for germination [39]. Subsequently, WPM supplemented with 0.2 mg·l^−1^ TDZ and 0.1 mg·l^−1^ 3-indole butyric acid (IBA) was used to induce callus. The callus was then extensively proliferated using WPM supplemented with 1.0 mg·l^−1^ 2,4-dichlorophenoxyacetic acid (2,4-D) and 0.5 mg·l^−1^ naphthaleneacetic acid (NAA) [25]. We collected sufficient amounts of callus subjected to dark culture and light treatment (14 hours of light followed by 10 hours of darkness daily) for subsequent transcriptome sequencing as *in vitro* cultured materials. For *R. delavayi* (27°11′45″N, 106°1′5″E), similar to *R. bailiense*, we collected leaves adjacent to regular flowers and double-petaled flowers (which were close to wilting at the time of collection and thus not collected) as materials for subsequent transcriptome studies. Similarly, we also collected regular flowers and red patch flowers from *R. decorum* (27°13′44″N, 105°51′53″E), as well as regular flowers and double-petaled flowers from *R. agastum* (27°13′46″N, 105°51′16″E) for further research.

We also utilized public transcriptome resources of rhododendrons for analysis, including *R. delavayi* [40], *R. molle* [19], *R. decorum* (NCBI Project ID: PRJNA774925), *Rhododendron griersonianum* [41], *R. ‘Elsie Lee’* [42], *R. ‘Xiaotaohong’* [43], and *Rhododendron × pulchrum* [44]. For transcriptome resource information, please refer to Supplementary Table S1.

### Identification of Ca^2+^-ATPase at the pan-genome level, phylogenetic analysis, and functional enrichment analysis

The genomic resources of plants are sourced from public databases such as NCBI (https://www.ncbi.nlm.nih.gov/), China National Center for Bioinformation (https://www.cncb.ac.cn/), PlantGIR (http://plantgir.cn/) [45], and The Ericaceae Genome Resource Database (http://www.tegr.com.cn/) [46], among others. For genomic resource information, please refer to Supplementary Table S1.

Based on our previous research, we have improved the predictive accuracy of CAP genes [2]. First, we re-annotated the functions of the genomes of all species using eggnog-mapper2 (default parameter) [47]. From the newly annotated files, we precisely filtered the CAP genes in each genome using the EC number ‘3.6.3.8’. These CAP genes corresponded accurately to the KEGG_ko number ‘K01537’, which is the exclusive number for ‘P-type Ca^2+^ transporter type 2C’. Furthermore, these CAPs corresponded to the PFAM structure ‘Cation_ATPase’ and the description ‘magnesium-dependent enzyme catalyzes the hydrolysis of ATP coupled with the transport of calcium’. To ensure the robustness of our findings, Batch CD-Search (https://www.ncbi.nlm.nih.gov/Structure/bwrpsb/bwrpsb.cgi) was employed to verify the structural domains identified within the candidate gene set. This method enabled the acquisition of a reliable and precise collection of CAP gene family members for further analysis. The phylogenetic analysis of CAPs was conducted using the MAFFT (parameter: --auto) and IQtree (parameter: -m MFP -bb 5000) workflow; for more details, please refer to Supplementary Note S7.

### Distribution and phylogenetic analysis of rhododendrons

The distribution area of *Rhododendron* species was obtained from the Global Biodiversity Information Facility (https://www.gbif.org/). China’s soil distribution data were sourced from the National Earth System Science Data Center (http://soil.geodata.cn/). China’s elevation map was obtained from the Resource and Environmental Science Data Platform (https://www.resdc.cn/Default.aspx). These datasets were combined and analyzed using ArcGIS v10.8.1 (https://www.esri.com/en-us/arcgis/products/arcgis-pro/overview) for visualization. The construction of the species tree was conducted using the Orthofinder-MCMCtree pipeline; for further details, please refer to Supplementary Note S8.

### CAPs duplication pattern, Ka/Ks calculation, and synteny analysis

We firstly investigated genome-wide patterns of gene duplication. Gene duplications were annotated using DupGen_finder (default parameters) [48] across five duplication models: tandem duplication (TD), WGD, proximal duplication (PD, defined as genes separated by fewer than 10 loci on the same chromosome), transposed duplication (TRD), and dispersed duplication (DSD). Then, the duplication patterns of CAPs were extracted from the results obtained in the previous step. Subsequently, synonymous substitution rates (Ks), nonsynonymous substitution rates (Ka), and Ka/Ks ratios were calculated for CAP pairs using KaKs_Calculator v2 [49]. Local gene synteny across species was analyzed using the ‘jcvi.graphics.synteny’ feature of MCscanX [50]. Data visualization and significance analysis were conducted using R-4.1.3 (https://cran.r-project.org/index.html) and Rstudio V1.2.5042 (https://posit.co/products/open-source/rstudio/). For the detection of copy number variation of CAP-a on Chr2 in the *R. delavayi* population (resource sourced from NCBI-BioProject: PRJNA659608), BWA v0.7.18 [51] was first utilized to map the genomic sequencing data of each individual to the *R. delavayi* genome. Subsequently, CNVkit v0.9.12 [52] was employed to detect the copy number variations of CAP-a in each individual.

### Transcriptome research

In this study, we conducted transcriptome sequencing on different tissue samples of *R. bailiense*, *R. delavayi*, *R. decorum*, and *R. agastum*. Total RNA was extracted using TRIzol (Invitrogen, California, United States) reagent, following the manufacturer’s instructions. The Illumina NovaSeq6000 system was employed to sequence the constructed cDNA library (2 × 150 bp). Subsequent assembly and analysis were conducted using the raw data.

All transcriptome data of rhododendrons involved in this study were analyzed and visualized using the following workflow. Low-quality bases, reads, and adapter sequences were removed using Trimmomatic version 0.39 [53]. Clean reads were then mapped to the respective *Rhododendron* genome assemblies using Parallel v20250222 (https://zenodo.org/records/14911163) in conjunction with HiSat2 (default parameters) [54]. For *Rhododendron* species without a genome, the transcriptome of *R. agastum* was mapped to the *R. delavayi* genome, as *R. agastum* is derived from a hybridization of *R. delavayi*, and their genotypes are closely related [55]. Similarly, the transcriptomes of *R. ‘Elsie Lee*’, *R. ‘Xiaotaohong*’, and *Rhododendron × pulchrum* were mapped to the *R. simsii* genome. The uniquely mapped reads were retained to calculate the counts and fragments per kilobase of transcript per million mapped fragments (FPKM) matrix using Stringtie v2.2.1 (default parameters) [56]. The visualization of transcriptome data was performed using the heatmap visualization tool in TBtools v2.057 [57]. For transcriptome resource information, please refer to Supplementary Table S1.

### Weighted gene co-expression network analysis

This study utilized the WGCNA_shiny plugin of TBtools [58] to perform WGCNA and visualization. The format was selected in ‘normalized count’ mode. To retain a larger number of genes, the Sample percentage, Expression Cutoff, and Reserved genes number were set to 0.1, 0.1, and 16 000, respectively. In the Module-net section, the parameters Min Module Size and Module Cuttree Height were set to their default values of 30 and 0.25, respectively. All other parameters were set to default. In selecting the primary co-expressed genes of CAPs, we chose approximately the top 150 genes based on weight ranking as the main co-expressed genes. Similar to the functional enrichment analysis of CAPs, we utilized the GO enrichment tool in TBtools v2.057 [57] to perform functional enrichment analysis on co-expressed genes, with the subset set to ‘default’. Terms that are significantly enriched with GO_level >3 are presented, including those from ‘Biological Process’, ‘Molecular Function’, and the main ‘Cellular Component’ categories, while redundant and obsolete terms are excluded.

### Three-dimensional structure prediction

We performed the three-dimensional (3D) structure prediction of CAPs and their interaction protein models using AlphaFold3 [59]. The ‘entity type’ parameter was configured to ‘protein’ mode. The ‘copy’ parameter was set to ‘1’. The reference structure for each protein was the best-ranked model (Rank 1). The visualization analysis of protein structures and the interactions between protein molecules was generated using PyMOL v3.1.3 [60].

### Gene cloning and plasmid construction

Specific primers were used to PCR amplify the CAPs, CIPKs, and CBL1 genes from the *R. bailiense* cDNA library (Supplementary Table S2). After electrophoresis of the PCR products, the corresponding gene products were excised and recovered from the gel. The CAPs, CIPKs, and CBL1 genes were then constructed into the p416GPD vector using the Seamless Cloning kit (Sangon Biotech, Shanghai, China). This vector contains the GPD promoter, a strong promoter in yeast that facilitates gene overexpression. The recombinant plasmids were transformed into *Escherichia coli* using the ice transformation method, and positive colonies were identified through colony PCR and Sanger sequencing. After identification, positive strains were cultured extensively to obtain high concentrations of plasmid.

### Yeast transformation and high calcium stress

The yeast strain BY4741 was utilized to express CAPs, CIPKs, and CBL1 for functional analysis. Yeast cells were transformed with one or more recombinant vectors using the PEG/LiAc method according to the Clontech Yeast Protocols Handbook [15]. Preliminary screening of yeast colonies was conducted on YPD plates supplemented with ampicillin. For yeast transformed with one vector, single-round colony PCR was employed to identify true positive colonies; for yeast transformed with three vectors, three-round colony PCR was used to confirm true positive colonies. The tolerance test for Ca^2+^ was performed by adding 300 mM CaCl_2_ to the YPD medium. To prevent precipitation of Ca^2+^ during autoclaving, a filter-sterilized 2 M CaCl_2_ solution was added to the YPD medium to achieve a final concentration of 300 mM Ca^2+^. It should be noted that an appropriate amount of HCl must be added to aid the dissolution of CaCl_2_. Positive yeast transformants were initially cultured overnight in liquid selective medium. To ensure consistent cell numbers across all spotting assays, the overnight yeast cell suspensions were centrifuged and normalized to an initial optical density OD_600_ of 1.0. After evenly aliquoting the yeast culture, a series of 10-fold dilutions (10^0^ to 10^−2^) were performed, followed by spotting on agar medium (YPD and YPD supplemented with 300 mM CaCl_2_). The cultures were incubated at 28°C for 2 days before imaging. The experiment was repeated with three independent transformants.

## Supplementary Material

Web_Material_uhag174

## Data Availability

The data underlying this article are available in the article and in its online supplementary material.
