# Supplementary material for: The evolution of Ca2+-ATPases across plants with profiles in Rhododendron and the function of key members in alleviating high calcium stress
Source: Hortic Res. 2026 Apr 29;13(9):uhag174. doi: 10.1093/hr/uhag174 (PMC13554806; doi:10.1093/hr/uhag174)
Supplement: Supplementary file 1 [file web_material_uhag174.zip › Supplementary_material5.pdf]

Supplementary Note S1. Analysis of CAPs' motifs in rhododendrons

The structure (Figure 2C) shown is that of autoinhibited CAPs, which have ten transmembrane proteins and four main structural domains: the actuator (A) domain (with a key motif of TGES), the nucleotide-binding (N) domain (with a key motif of KGASE), the phosphorylation (P) domain (with a key motif of DKTGT), and the AD domain at the N-terminus. For ER-type CAPs, the main difference from autoinhibited CAPs is the absence of the AD domain, resulting in a shorter N-terminus, while the other structural domains remain almost unchanged. We further selected *Rhododendron bailiense*, *R. delavayi*, and *R. simsii* to analyze the gene motifs of their CAPs (Supplementary Figure S2). The results indicate that the motifs of CAPs are relatively conserved; aside from some truncated member genes, most motifs of the same subfamily members are distributed in a regular and similar manner. The gene lengths of the vast majority of core members are approximately 1000 AAs. In addition, we found that the motif 1 located in the P-domain contains a conserved DKTGT motif; motif 3 located in the N-domain possesses a conserved KGAAE motif; and motif 6 located in the A-domain has a conserved TGE motif (Supplementary Figure S2B, C). The distribution of motifs suggests that the amino acid residues of CAPs exhibit a relatively conserved pattern, with the amino acid diversity of most motifs being not much high (Supplementary Figure S2B).

Supplementary Note S2. Functional enrichment analysis of different subfamilies of CAPs

To understand the functions of various subfamilies of CAPs, we conducted Gene Ontology (GO) enrichment analysis. In the GO annotations, the primary functions of CAP-a are described as "P-type calcium transporter activity" and "ATP hydrolysis activity" (Supplementary Figure S3A), which aligns with the general characteristics of CAPs. Regarding subcellular localization, CAP-a can be found on the plasma membranes (PMs) and organelle membranes (Supplementary Figure S4A), which is

consistent with previous studies on autoinhibited CAPs (i.e., ACAs) in Arabidopsis (Li et al., 2023). The functions of CAP-b are similar to those of CAP-a; however, the annotations for CAP-b additionally include terms such as "single fertilization" and "gametophyte development" (Supplementary Figure S3B), which corresponds to research on the involvement of autoinhibited CAPs in the pollen fertilization process (Rahmati Ishka et al., 2021). Similar to CAP-a, the subcellular localization of CAP-b is also primarily located at the plasma membrane (Supplementary Figure S4B). CAP-c is classified as an ER-type CAP (i.e., ECA) in the traditional classification of Arabidopsis (Rahmati Ishka et al., 2021). It is primarily located in the endoplasmic reticulum, Golgi apparatus, vacuolar membrane, and vesicles (Supplementary Figure S4B). Functionally, in addition to calcium transport enzyme activity, it possesses several noteworthy functions including "pyrophosphatase activity", "manganese ion transport", "root system development", and "response to cadmium ion" (Supplementary Figure S3B). The ability of ER-type CAPs to transport Mn^{2+} and Zn^{2+} has been previously verified (Costa et al., 2023). Although CAP-d is functionally annotated similarly to CAP-c (Supplementary Figure S3C, D), it is classified as autoinhibited CAPs in the traditional taxonomy of Arabidopsis (Rahmati Ishka et al., 2021). Its primary subcellular localization is also in the endoplasmic reticulum, but it additionally includes the inner membrane of chloroplasts and other organelle membranes (Supplementary Figure S4D). CAP-e and CAP-f, two subfamilies of CAPs unique to rhododendrons, show functional annotations indicating multiple stress response functions, such as "response to external biotic stimulus", among others (Supplementary Figure S3E, F). Regarding localization, the results indicate that these two CAPs are similarly localized primarily in vacuoles, chloroplasts, and the Golgi apparatus (Supplementary Figure S4E, F).

Supplementary Note S3. Regional characteristics of distribution of different *Rhododendron* species

The geographical features of the mountainous regions have led to the

isolation-driven evolution of rhododendrons, resulting in numerous unique species, some of which exist only on a few mountain peaks. In ancient China, during the establishment of provinces, there were significant geographical boundaries between the southwestern provinces (Figure 3B, D); for example, the Wumeng Mountain Range separates Guizhou from Yunnan. Guizhou is the only province in China without plains, while Guizhou, Yunnan, and Sichuan are filled with various mountain ranges, creating favorable conditions for the radiation evolution of rhododendrons (Ma et al., 2022). The soils in the southwestern mountainous region of China generally exhibit low nutrient characteristics (low phosphorus and nitrogen), whereas the soils in low-altitude areas display higher nutrient characteristics (high phosphorus and nitrogen) (Xia et al., 2022). In such situations, the genome sequencing samples of rhododendrons from different provinces exhibit distinct characteristics (Figure 3C). The differences in topography inevitably lead to variations in the growth soil, which is why we have also mapped the distribution of rhododendrons in relation to soil (Figure 3B). The core soils in southern China include yellow earths, red earths, and paddy soil. Rhododendrons typically prefer acidic soils such as red earths and yellow earths, and most of them are distributed in these areas. Unique occurrences are also found in Guizhou, as this region boasts the most extensive karst topography in China, characterized by limestone soils associated with karst formations. In this context, many *Rhododendron* species adapted to the karst landscape have emerged, with the *R. bailiense* being the most typical example (Wen et al., 2025, Ma et al., 2015). The distribution of different *Rhododendron* species exhibits distinct regional characteristics (Figure 3B). For instance, *R. molle* is primarily found in low-altitude areas, while species such as *R. delavayi* is exclusively present in relatively high-altitude regions. Geographic and geomorphological features influence the formation of *Rhododendron* species, shaping their inherent characteristics (Xia et al., 2022).

Supplementary Note S4. The environment of Guizhou shapes a comparative case of high CAPs copies in *R. bailiense*

A pronounced comparative case is observed between *R. bailiense* collected from BRNR and *R. molle* collected from eastern Hunan, as well as *R. williamsianum* collected from western United States, which show significant differences in CAPs copy number (Figure 2B). Furthermore, another notable difference is observed in the CAPs copy number between *R. bailiense* and *R. prattii* from western Sichuan, as well as *R. griersonianum* from western Yunnan (Figure 2B). Although these three species of *Rhododendron* are geographically close (Figure 3C), there are still significant differences in the number of CAPs copies among them.

Supplementary Note S5. Analysis of CAPs expression in various rhododendrons

In *R. simsii*, the expression patterns at the subfamily level exhibit characteristics similar to those in *R. bailiense* (Figure 5B). Notably, *Rhsim08G0094100* (CAP-d) is highly expressed during the T1 stage of flower development. This is similar to the expression patterns of ACA2 and ACA7 localized in the endoplasmic reticulum in Arabidopsis, which regulate calcium signaling-mediated pollen fertility maturation (Rahmati Ishka et al., 2021), suggesting that *Rhsim08G0094100* possesses the same function. In *R. decorum*, aside from the overall expression patterns of CAPs seen in *R. bailiense*, a noteworthy point is that *Rde03G034180* (CAP-b) and *Rde08G009950* (CAP-d) show obviously elevated expression in trees at mid and high altitudes (Figure 5C), indicating that these two specific CAPs are regulated by altitude-related environmental factors. This finding aligns with the high expression of CAP-b and CAP-d in *R. bailiense* at high altitudes. Furthermore, when using *in-vitro*-cultured seedling materials, we found that regardless of the altitude of the trees, there were no significant differences in CAPs expression among the offspring (Figure 5C), indicating that the differences in CAPs expression in trees arise from variations in their surrounding living environments rather than individual effects or genetic

differences. Additionally, altering the pH in the *in vitro* culture environment did not significantly change the expression of CAPs (Figure 5C), indicating that CAPs are not sensitive to changes in pH.

We further investigated the expression of CAPs under various conditions in *R. delavayi* (Figure 6A). The overall expression pattern at the subfamily level of CAPs in *R. delavayi* is generally consistent with that of CAPs in other species of rhododendrons. Notably, *Rhdel03G0341700* (CAP-b) and *Rhdel08G0101000* (CAP-d) are highly expressed in floral organs such as the spotted throat and spotted petals, which may be related to floral development, similar to the findings in *R. simsii*. Under drought treatment conditions, *Rhdel03G0341700*, *Rhdel08G0101000*, and *Rhdel13G0100100* showed high expression on the 9th day of treatment (Figure 6A), analogous to the trend of enhanced drought tolerance associated with vacuolar localization of ACA11 in Arabidopsis (Su et al., 2024). In *in-vitro* leaf conditions, we noted that some CAPs (e.g., *Rhdel02G0039600*) were induced early by TDZ (Thidiazuron), reflecting the specific hormonal induction effects on CAPs.

Furthermore, we observed variations in CAPs under more diverse conditions in additional *Rhododendron* species (Supplementary Figures S7, S8). Apart from the overall expression pattern classified by subfamily, which is essentially the same as that of *R. bailiense*, the following situations are also noteworthy: In *R. griersonianum*, the pH changes did not significantly alter the expression of CAPs (Supplementary Figure S7A). In *R. 'Xiaotaohong'*, initial infection by *Alternaria* sp. strain caused an increase in the expression of genes such as *Rhsim03G0267700* (CAP-b) and *Rhsim08G0094100* (CAP-d) (Supplementary Figure S8B). Notably, in *R. 'Elsie Lee'*, cold acclimation and winter conditions resulted in an increase in the expression of *Rhsim03G0267700* (CAP-b), indicating that it is a gene regulated by cold (Supplementary Figure S8A).

Supplementary Note S6. Analysis of Co-expression Networks of CAPs in various *Rhododendron* species.

In addition to the *R. bailiense*, to further understand the co-expression networks of CAPs in other *Rhododendron* species, we conducted WGCNA analysis on the transcriptome data of more *Rhododendron* species (Supplementary Figure S12). The correlation plots between gene expression modules and different conditions are presented in Supplementary Figures S13-S15. Briefly, similar to the high expression of CAP-b corresponding to the MEyellow module in the stem of *R. bailiense*, we focused on the modules corresponding to the high expression of CAP-b and CAP-d under various conditions in different *Rhododendron* species. For example, *Rhdel03G0341700* (CAP-b) is highly expressed in the unspotted petals of *R. delavayi*, corresponding to the MEbrown module (Supplementary Figure S13). Supplementary Figure S18 shows a positive correlation between the genes in the relevant modules and the corresponding conditions or tissue types. For the genes in these relevant modules, like the corresponding CAPs, they are mostly highly expressed under the respective conditions (tissue types or environmental conditions) (Supplementary Figure S11). Enrichment analysis of the MEgreen module genes in *R. bailiense* revealed that the genes are enriched in terms such as "chloroplast", "catabolic process", and "response to oxidative stress" (Supplementary Figure S16A; Supplementary Data Set S2). Similarly, the enrichment analysis of the MEbrown module in *R. delavayi* involved terms like "transport" and "anion binding" (Supplementary Figure S16B), indicating frequent material transport in this process. The enrichment analysis of the MEpink module in *R. 'Elsie'* (associated with winter environments) included terms such as "chloroplast" and "response to light stimulus" (Supplementary Figure S16C), which, like *R. bailiense*, also involved changes in chloroplast-related proteins that may be associated with leaf senescence. Similarly, the enrichment analyses of the other three modules (MEblack in *R. delavayi*, MEblack in *R. decorum*, and MElightcyan in *R. simsii*) involved numerous genes related to stress response (Supplementary Figure S17), indicating that the expression of CAPs is

generally accompanied by an increase in the expression of stress response-related genes.

For *Rb12G0003510* (CAP-d) in *R. bailiense*, we constructed its co-expression network (Supplementary Figure S16D). There are several genes similar to those studied in *Rb11G0000068*, such as *Rb03G0001156* (Calmodulin-binding receptor-like cytoplasmic kinase 3) and *Rb04G0003670* (Serine-Threonine-protein kinase). However, there are also some unique genes associated with the expression of *Rb12G0003510*, such as *Rb09G0005275* (Abscisic Acid-Insensitive 5-like protein) and *Rb13G0003439* (RING-type E3 ubiquitin transferase), which are involved in abscisic acid response and ubiquitination processes, and may require special attention. The expression analysis indicates that the genes involved in Supplementary Figure S16D are highly expressed in stems and old leaves (Supplementary Figure S16E). Enrichment analysis shows that the co-expressed genes of *Rb11G0000068* and *Rb12G0003510* are involved in numerous stress responses and hormone responses (Supplementary Figure S19), with *Rb12G0003510* also associated with co-expression of genes related to developmental regulation.

To further identify some core co-expressed genes of CAPs, we utilized Venn diagrams to examine the common co-expressed genes of CAP-b and CAP-d across different species. For CAP-b, the high-expression gene common to all four *Rhododendron* species is "Mitochondrial carrier," suggesting that the expression of CAP-b is associated with increased energy metabolism (Supplementary Figure S16G). Additionally, noteworthy co-expressed genes found in three *Rhododendron* species include "E3 ubiquitin ligase," "Glycosyl hydrolase 17," and "Universal stress protein." In the analysis of CAP-d, we found that all four *Rhododendron* species involved the "E3 ubiquitin transferase" (Supplementary Figure S16H), indicating that the E3 ubiquitin-mediated protein degradation process may be active when CAP-d is highly expressed. We hypothesize that E3 ubiquitination may tend to degrade excessive stress response-related proteins, warranting further investigation. Furthermore, other noteworthy co-expressed genes in three rhododendron species include "phosphatase," "Ras-related protein," and "Zinc finger-C3HC4." In the co-expression networks of

Rb11G0000068 and *Rb12G0003510*, both MYB and ERF (Ethylene-responsive transcription factor) transcription factors are involved (Figure 7F and Supplementary Figure S16D). Therefore, we speculate that the MYB and ERF presented may directly regulate the expression of *Rb11G0000068* and *Rb12G0003510*. Through promoter analysis, we identified numerous binding sites for MYB and ERF (Supplementary Figure S16F). Among them, MYB belongs to the REVEILLE type, which has been shown to regulate the circadian clock and respond to temperature, but further research is still needed (Tian et al., 2022).

Supplementary Note S7. Phylogenetic analysis of CAPs

For the phylogenetic analysis of CAPs, the protein sequences of the CAPs were first extracted from the CAPs' IDs obtained in the previous step. Protein sequence alignment was conducted with MAFFT v7.486 (default parameters) (Nakamura et al., 2018). A maximum likelihood phylogenetic tree was constructed using IQtree v2.2.0 (Minh et al., 2020), employing ModelFinder ("-m MFP") and ultrafast bootstrapping ("-bb 5000"). The resulting trees were refined through iTOL (Letunic and Bork, 2021). Conservative motifs within CAPs were detected via MEME (Bailey et al., 2015), employing a motif limit of 10 under default settings. For the functional enrichment and subcellular localization enrichment analysis of different subfamilies of CAPs, the GO enrichment tool of TBtools v2.057 (Chen et al., 2023) was utilized. The subset was set to "default". Terms with GO_level > 3 that were significantly enriched are presented, while redundant and obsolete terms were excluded.

Supplementary Note S8. Species phylogenetic analysis of rhododendrons

We constructed a species phylogenetic tree using protein sequences from representative species of Rosids, Asterids, Cornales, and Ericales, including the genus *Rhododendron*. This represents an advancement over our previous research (Wen et al., 2025), incorporating recently genome-sequenced *Rhododendron* species: *R.*

liliiflorum, *R. concinnum*, *R. vialii*, *R. prattii*, and *R. platypodum*. OrthoFinder v2.5.4 (Emms and Kelly, 2019) was employed to identify 55,069 orthogroups from the input sequences using default parameters. Among these, 584 low-copy orthologues were detected as single-copy genes present in at least 80% of species within each orthogroup. The software also generated an initial phylogenetic tree, which was subsequently refined to estimate species divergence times. Single-copy gene sequences were extracted and aligned using MUSCLE v5.1 (default parameters) (Edgar, 2004), followed by trimming with trimAl v1.4 (trimal -gt 0.8 -st 0.001 -cons 60) (Capella-Gutierrez et al., 2009). The resulting species tree from OrthoFinder and the sequence alignment file were integrated into MCMCtree (part of the PAML package, v4.10) (Yang, 2007) to construct the final phylogenetic tree and infer timeline (using the 'JC69' model, with other parameters set to default). Calibration points for the analysis included one soft bound and two fossil records across three nodes: (1) Asterids-Rosids divergence (116–126 Million years ago, Mya) (Li et al., 2019), (2) the crown node of Ericales (89.8 Mya) (Nixon and Crepet, 1993), and (3) the stem node of *Rhododendron* (56 Mya) (Collinson and Crane, 1978).

Reference

1. Bailey T.L., Johnson J., Grant C.E. & Noble W.S. 2015. The MEME Suite. ***Nucleic Acids Research,*** 43, W39-49.
2. Capella-Gutierrez S., Silla-Martinez J.M. & Gabaldon T. 2009. trimAl: a tool for automated alignment trimming in large-scale phylogenetic analyses. ***Bioinformatics,*** 25, 1972-1973.
3. Chen C., Wu Y., Li J., Wang X., Zeng Z., Xu J., Liu Y., Feng J., Chen H., He Y. & Xia R. 2023. TBtools-II: A "one for all, all for one"bioinformatics platform for biological big-data mining. ***Molecular Plant,*** 16, 1733-1742.
4. Collinson M.E. & Crane P.R. 1978. RHODODENDRON SEEDS FROM PALAEOCENE OF SOUTHERN ENGLAND. ***Botanical Journal of The Linnean Society,*** 76, 195-205.
5. Costa A., Resentini F., Buratti S. & Bonza M.C. 2023. Plant Ca(2+)-ATPases: From biochemistry to signalling. ***Biochim Biophys Acta Mol Cell Res,*** 1870, 119508.
6. Edgar R.C. 2004. MUSCLE: multiple sequence alignment with high accuracy and high throughput. ***Nucleic Acids Research,*** 32, 1792-1797.
7. Emms D.M. & Kelly S. 2019. OrthoFinder: phylogenetic orthology inference for comparative genomics. ***Genome Biology,*** 20.
8. Letunic I. & Bork P. 2021. Interactive Tree Of Life (iTOL) v5: an online tool for phylogenetic tree display and annotation. ***Nucleic Acids Research,*** 49, W293-W296.
9. Li H.T., Yi T.S., Gao L.M., Ma P.F., Zhang T., Yang J.B., Gitzendanner M.A., Fritsch P.W., Cai J., Luo Y., Wang H., Van Der Bank M., Zhang S.D., Wang Q.F., Wang J., Zhang Z.R., Fu C.N., Yang J., Hollingsworth P.M., Chase M.W., Soltis D.E., Soltis P.S. & Li D.Z. 2019. Origin of angiosperms and the puzzle of the Jurassic gap. ***Nature Plants,*** 5, 461-470.
10. Li Z., Harper J.F., Weigand C. & Hua J. 2023. Resting cytosol Ca²⁺ level maintained by Ca²⁺ pumps affects environmental responses in Arabidopsis. ***Plant Physiology,*** 191, 2534-2550.
11. Ma Y., Chamberlain D., Sun W. & Zhang C. 2015. A new species of *Rhododendron* (Ericaceae) from Baili *Rhododendron* nature reserve, NW Guizhou, China. ***Phytotaxa,*** 195, 197.
12. Ma Y., Mao X., Wang J., Zhang L., Jiang Y., Geng Y., Ma T., Cai L., Huang S., Hollingsworth P., Mao K., Kang M., Li Y., Yang W., Wu H., Chen Y., Davis C.C., Shrestha N., Ree R.H., Xi Z., Hu Q., Milne R.I. & Liu J. 2022. Pervasive hybridization during evolutionary radiation of *Rhododendron* subgenus *Hymenanthes* in mountains of southwest China. ***National Science Review,*** 9, nwac276.
13. Minh B.Q., Schmidt H.A., Chernomor O., Schrempf D., Woodhams M.D., Von Haeseler A. & Lanfear R. 2020. IQ-TREE 2: New Models and Efficient Methods for Phylogenetic Inference in the Genomic Era. ***Molecular Biology and Evolution,*** 37, 1530-1534.

14. Nakamura T., Yamada K.D., Tomii K. & Katoh K. 2018. Parallelization of MAFFT for large-scale multiple sequence alignments. ***Bioinformatics,*** 34**,** 2490-2492.
15. Nixon K.C. & Crepet W.L. 1993. Late Cretaceous Fossil Flowers Of Ericalean Affinity. ***American Journal of Botany,*** 80**,** 616-623.
16. Rahmati Ishka M., Brown E., Rosenberg A., Romanowsky S., Davis J.A., Choi W.G. & Harper J.F. 2021. Arabidopsis Ca2+-ATPases 1, 2, and 7 in the endoplasmic reticulum contribute to growth and pollen fitness. ***Plant Physiol,*** 185**,** 1966-1985.
17. Su J., He B., Li P., Yu B., Cen Q., Xia L., Jing Y., Wu F., Karnik R., Xue D., Blatt M.R. & Wang Y. 2024. Overexpression of tonoplast Ca(2+)-ATPase in guard cells synergistically enhances stomatal opening and drought tolerance. ***J Integr Plant Biol,*** 66**,** 1587-1602.
18. Tian Y.-Y., Li W., Wang M.-J., Li J.-Y., Davis S.J. & Liu J.-X. 2022. REVEILLE 7 inhibits the expression of the circadian clock gene EARLY FLOWERING 4 to fine-tune hypocotyl growth in response to warm temperatures. ***Journal of Integrative Plant Biology,*** 64**,** 1310-1324.
19. Wen S., Cai X., Yang K., Hong Y., Fan F., Wang Q., Zhang B., Hou Q., Leng Y., Qiao G., Wen X. & Shen X. 2025. Chromosome-level genome assembly of a rare karst-growing Rhododendron species provides insights into its evolution and environmental adaptation. ***Journal of Systematics and Evolution,*** 63**,** 245-267.
20. Xia X.M., Yang M.Q., Li C.L., Huang S.X., Jin W.T., Shen T.T., Wang F., Li X.H., Yoichi W., Zhang L.H., Zheng Y.R. & Wang X.Q. 2022. Spatiotemporal Evolution of the Global Species Diversity of *Rhododendron*. ***Molecular Biology And Evolution,*** 39**,** msab314.
21. Yang Z.H. 2007. PAML 4: Phylogenetic analysis by maximum likelihood. ***Molecular Biology And Evolution,*** 24**,** 1586-1591.

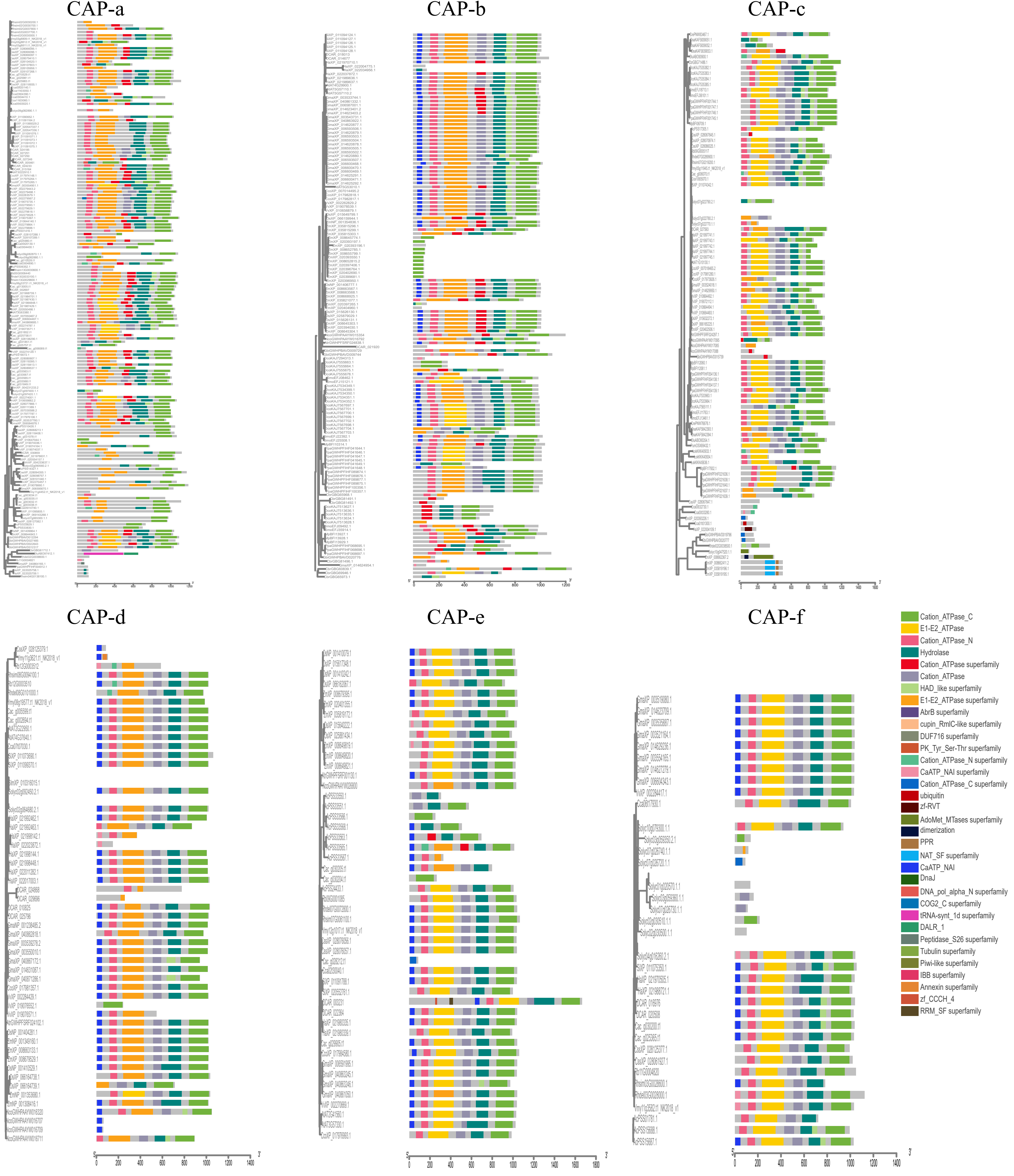

Supplementary Figure S1. Structural domain composition characteristics of CAP-a to CAP-f.

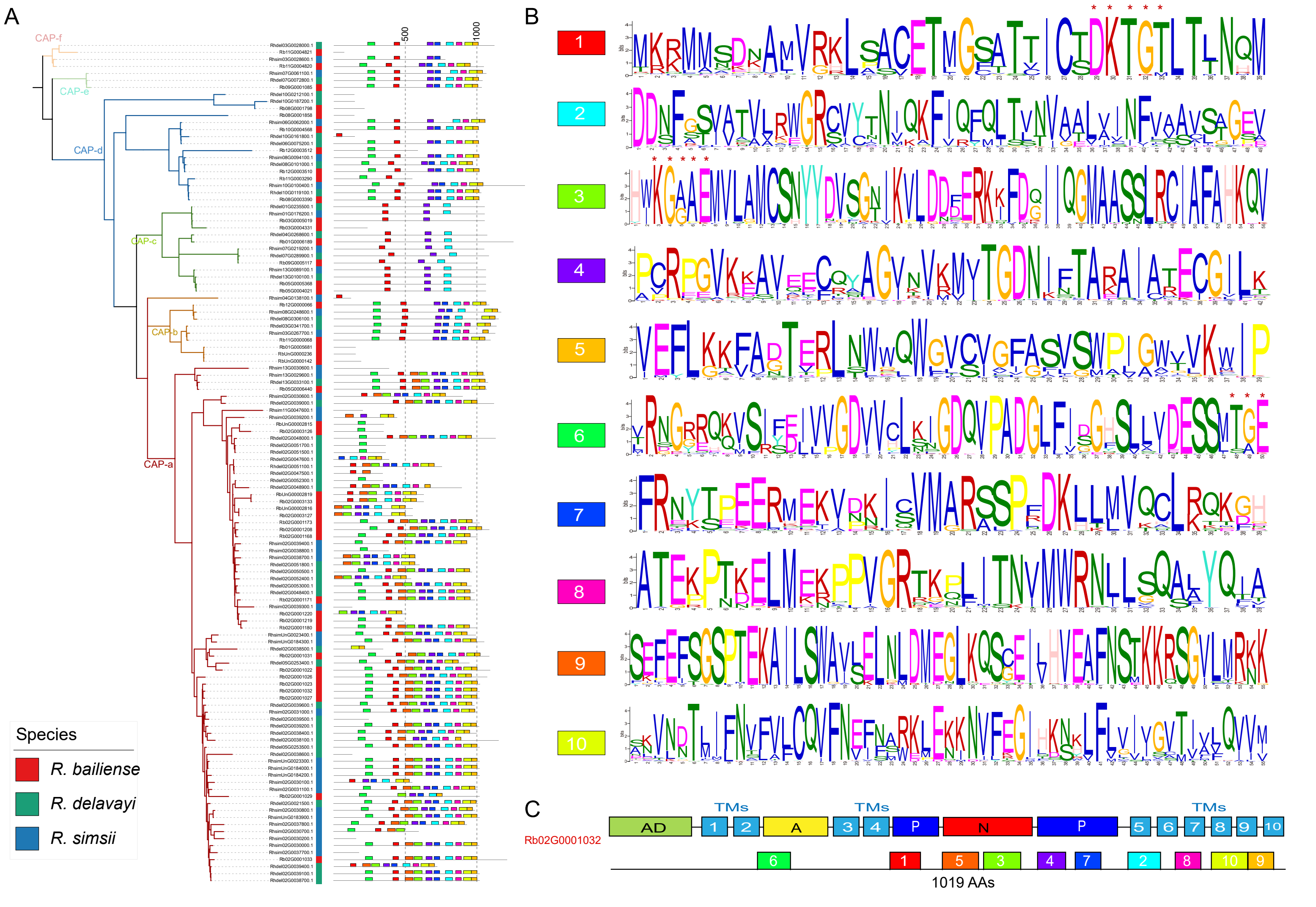

Supplementary Figure S2. Analysis of CAPs' motifs in *Rhododendron*. (A) Using *R. bailiense*, *R. delavayi*, and *R. simsii* as representatives, the distribution information of CAPs ’ motifs is presented. Most CAPs are approximately 1000 amino acids in length. (B) The amino acid composition information of the 10 motifs. Red asterisks highlight the conserved DKTGT motif of the P-domain, the conserved KGAAE motif of the N-domain , and the conserved TGE motif of the A-domain. (C) Taking *Rb02G0001032* as an example, the figure shows the correspondence between CAP domains and motifs.

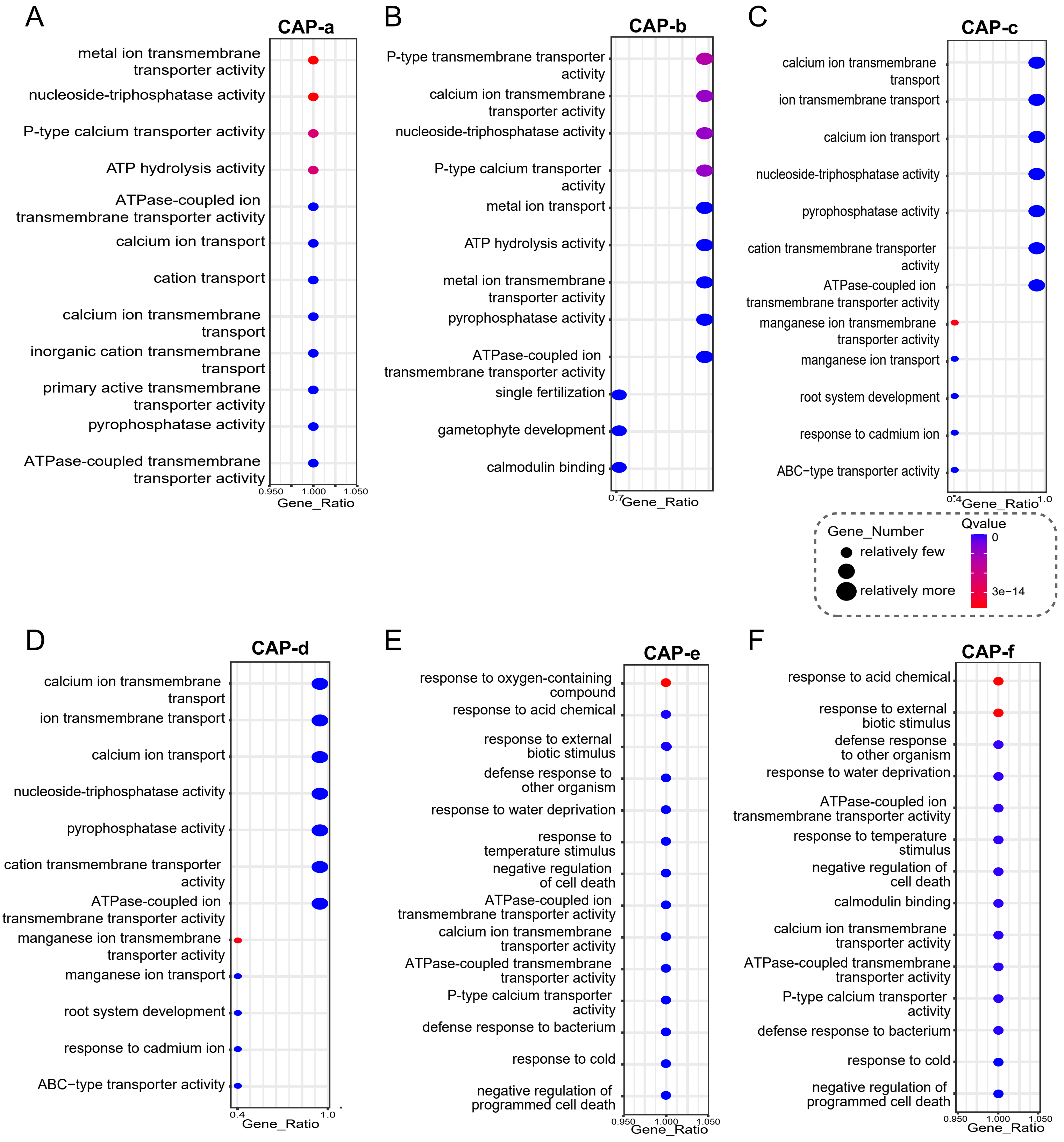

Supplementary Figure S3. Functional enrichment analysis of CAPs. (A-F) GO functional enrichment analysis of CAP-a to f, including terms related to "Molecular Function" and "Biological Process." Terms with GO_level > 3 and significantly enriched terms are presented, while some redundant and outdated terms have been excluded.

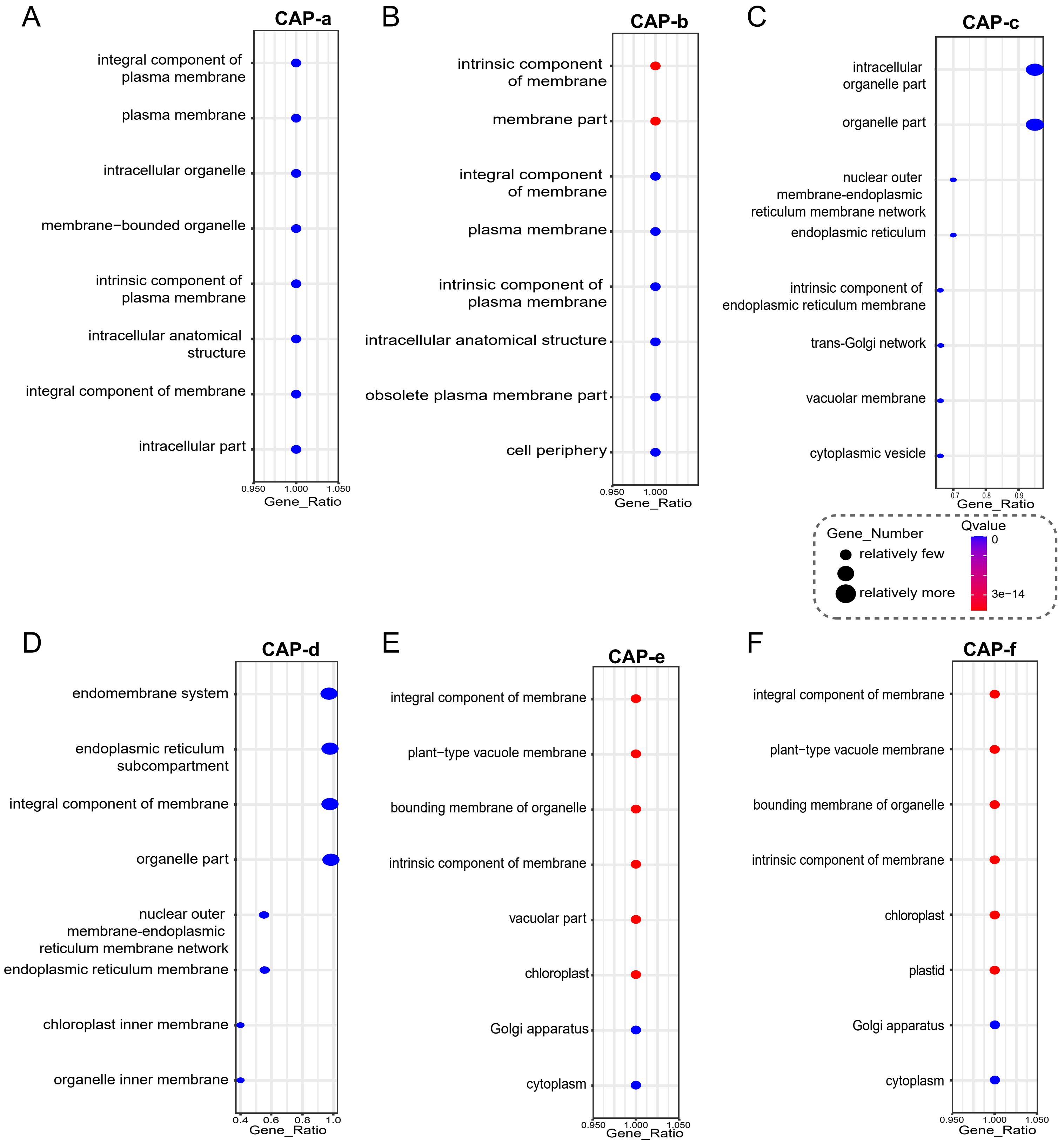

Supplementary Figure S4. Subcellular enrichment analysis of CAPs. (A-F) GO functional enrichment analysis of CAP-a to f, including terms related to "Cellular Component." Terms with GO_level > 3 and significantly enriched terms are presented, while some redundant and outdated terms have been excluded.

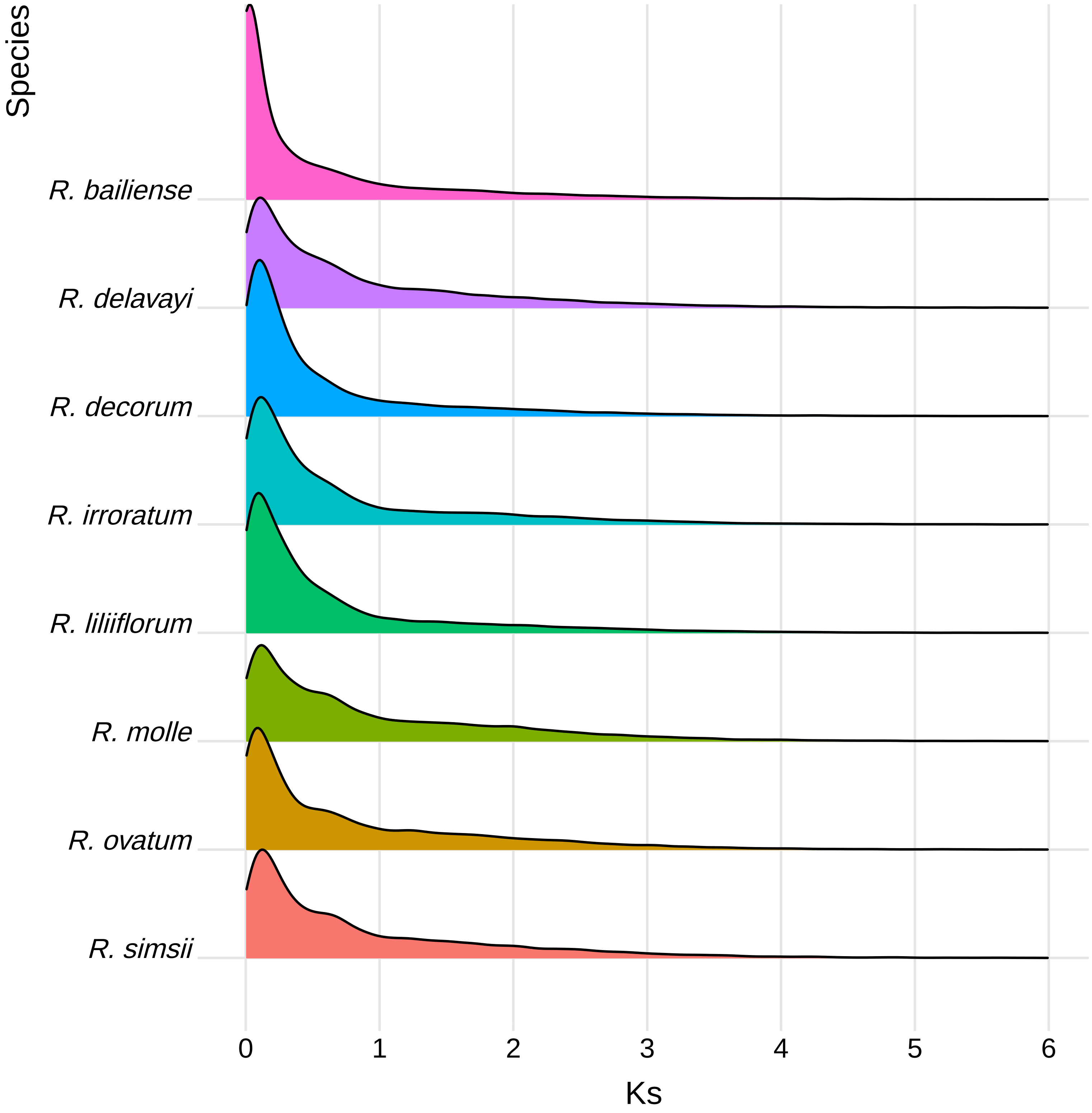

Supplementary Figure S5. The total Ks distribution of all gene pairs among eight species of *Rhododendron*.

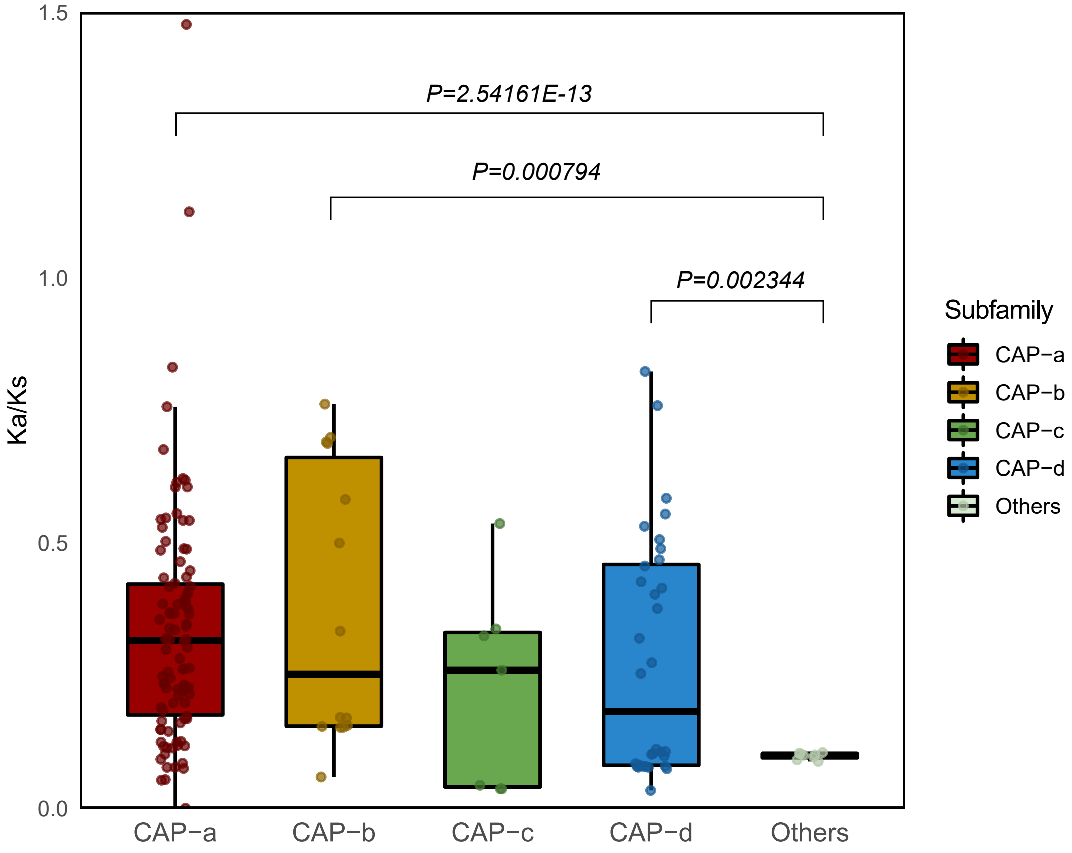

Supplementary Figure S6. The boxplot displays the comparison of the nonsynonymous substitution rate (Ka) to the synonymous mutation rate (Ks) for different CAP gene pairs. The datasets with significant differences are marked with P-values ($P < 0.05$), and the statistical method used is the T-test.

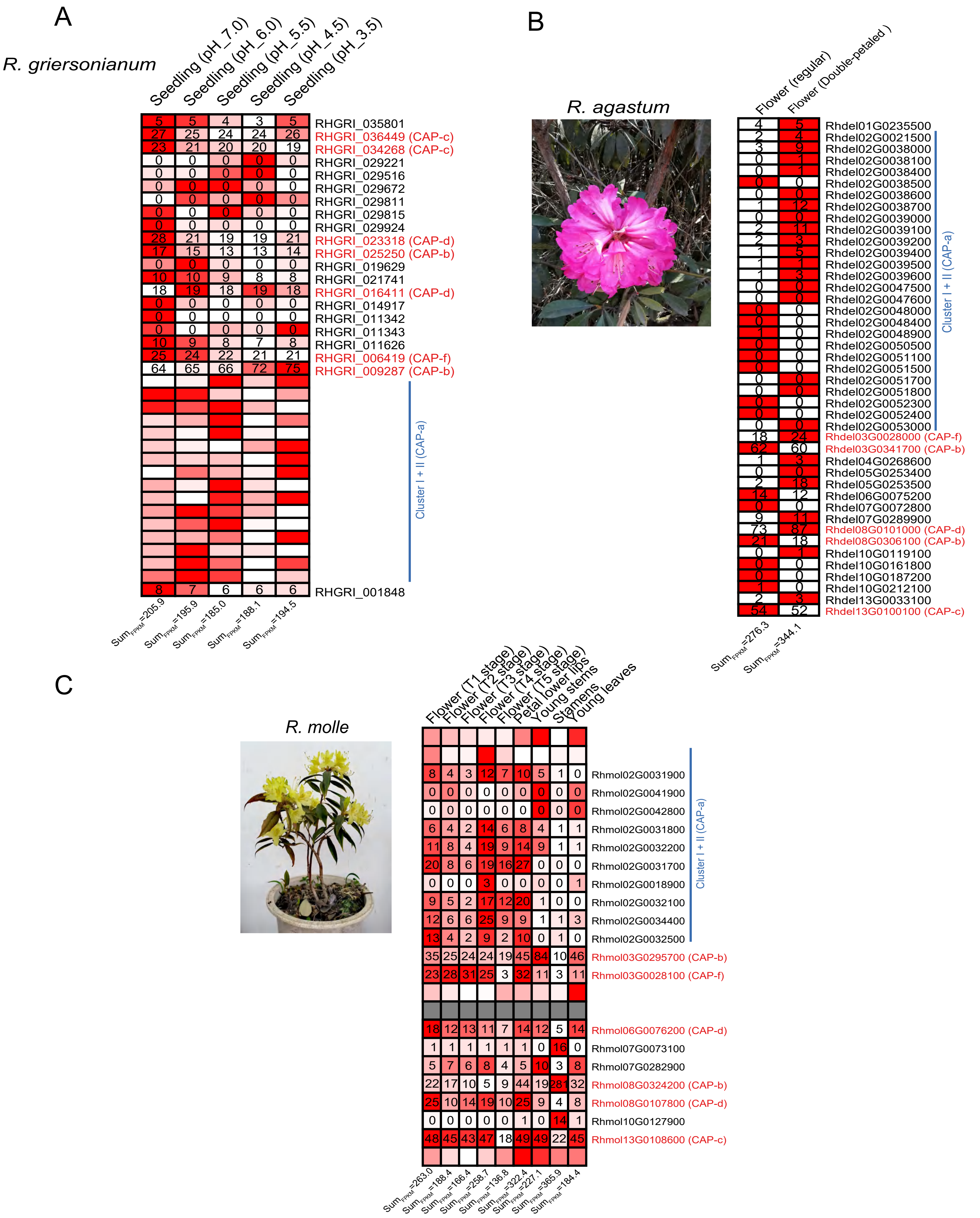

Supplementary Figure S7. Expression patterns of CAPs under more conditions in other species. (A-C) Plant phenotypes and expression patterns of CAPs in various tissues of *R. griersonianum*, *R. agastum*, and *R. molle*. The values in the boxes represent the FPKM values of gene expression. CAPs with very low expression in any case are not identified with FPKM values (< 1). Most CAP -a genes (mainly belonging to Cluster I + II) are at low expression levels. The total FPKM values of CAPs for each condition are annotated below the heatmap, revealing the overall levels of CAPs expression under these conditions.

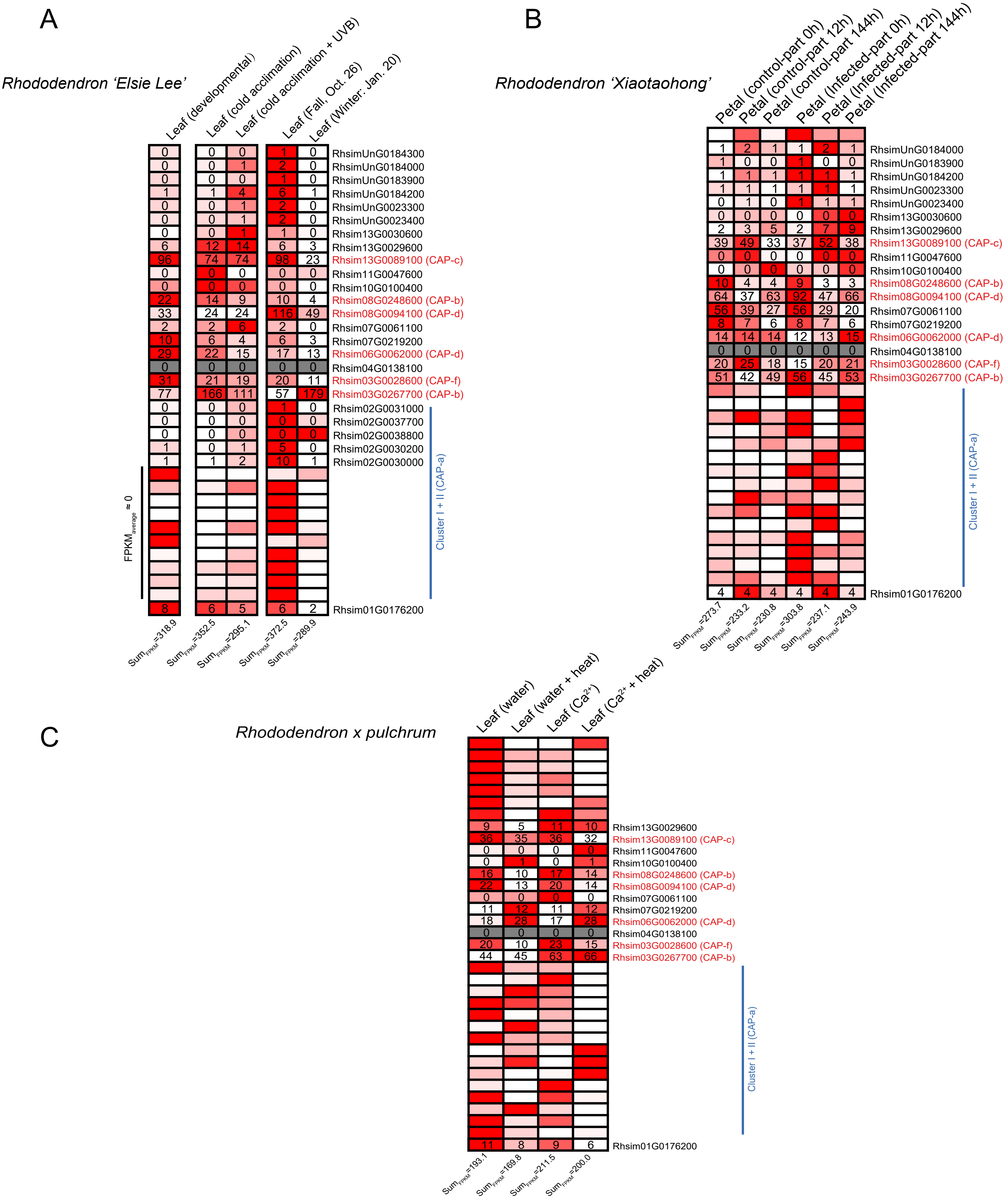

Supplementary Figure S8. Expression patterns of CAPs under other abiotic stress conditions. (A-C) The expression patterns of CAPs in *R. 'Elsie Lee'*, *R. 'Xiaotaohong'*, and *Rhododendron x pulchrum* under different abiotic stress treatments. The numbers in the squares represent the FPKM values of gene expression. CAPs with expression levels that are consistently low are not identified with FPKM values (< 1). Most CAP-a genes (mainly belonging to Cluster I + II) are at low expression levels. The total FPKM values of CAPs for each condition are annotated below the heatmap, revealing the overall level of CAPs expression under those conditions.

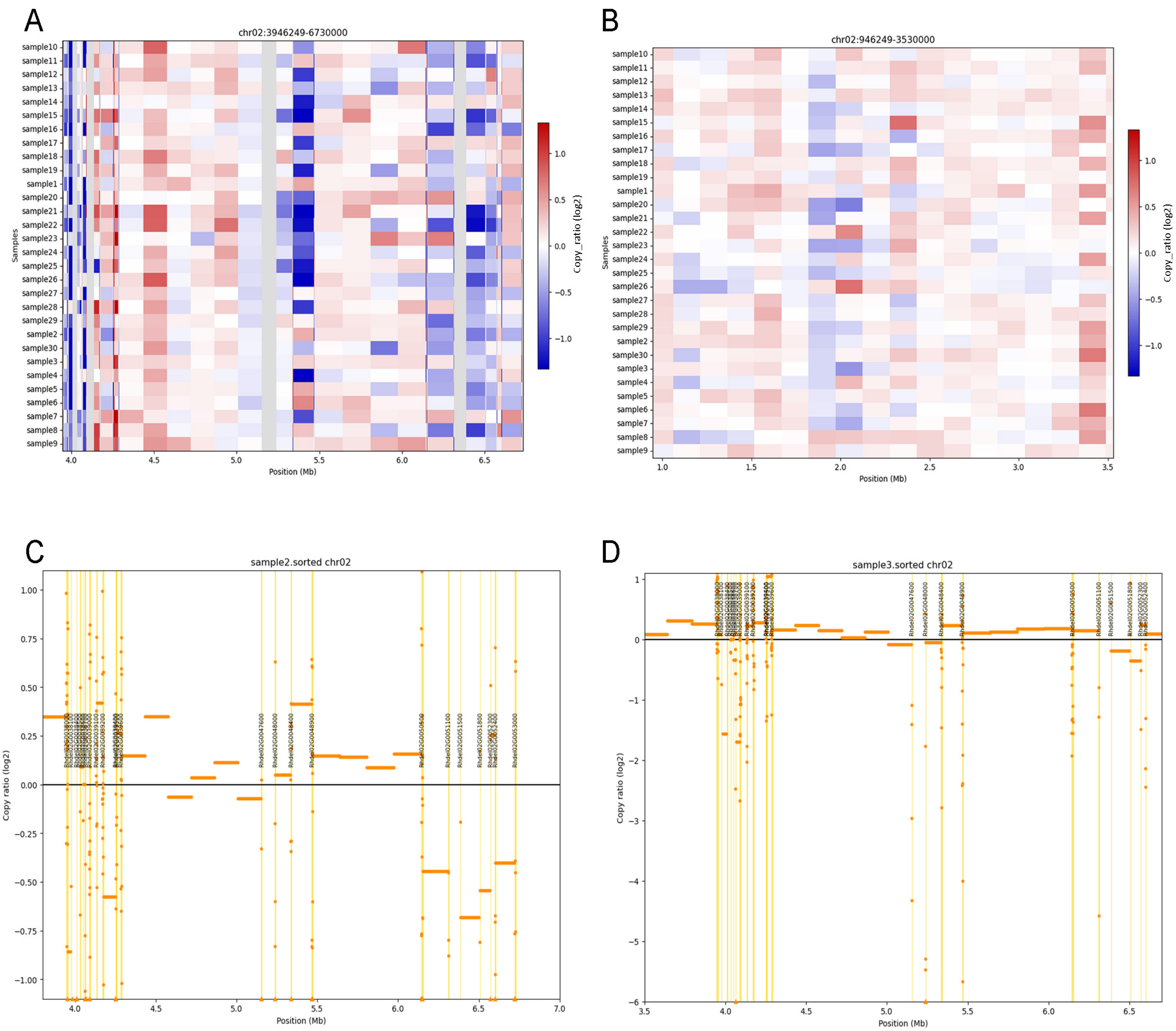

Supplementary Figure S9. 30 resequenced materials of *R. delavayi* reveal high-frequency variability in the CAP-a gene cluster. (A) The heatmap illustrates the high-frequency copy number variations of the CAP-a gene cluster on Chr2 (3.94 - 6.73 Mb) across the 30 resequenced *R. delavayi* materials. (B) A heatmap of gene copy number variations in the region near the CAP-a gene cluster on Chr2 (0.94 - 3.53 Mb) is shown for comparison. The heatmap indicates that the frequency of copy number variations in this region is lower than that of the CAP-a gene cluster. (C and D) The copy number variation profiles of CAP-a members from two resequenced samples are exemplified. The figure illustrates that these two samples exhibit high-frequency and unstable copy number variations at the CAP-a gene loci on Chr2, with both the magnitude and instability exceeding those at adjacent loci. The yellow lines and labels indicate the CAP-a gene loci, specifically the CAP-a gene cluster on Chr2.

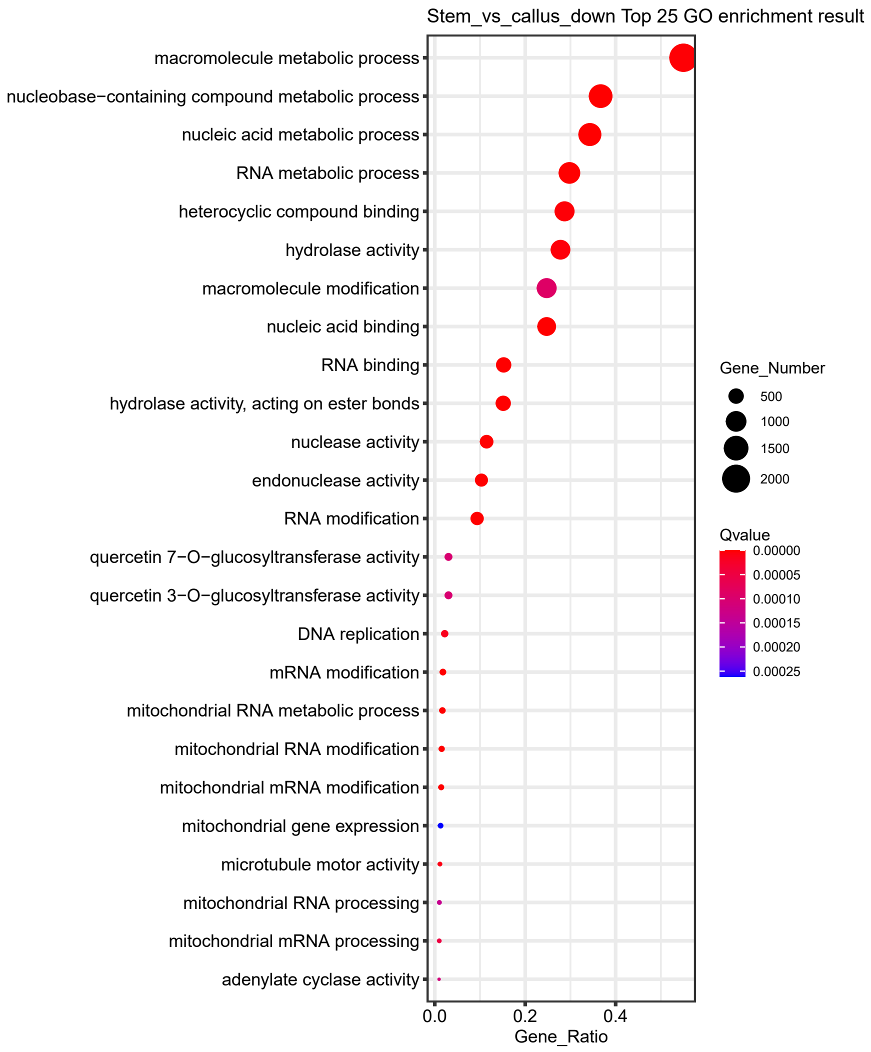

Supplementary Figure S10. The GO enrichment analysis illustrates the functional information of the downregulated expressed genes in the stems of *R. bailiense* compared to the callus.

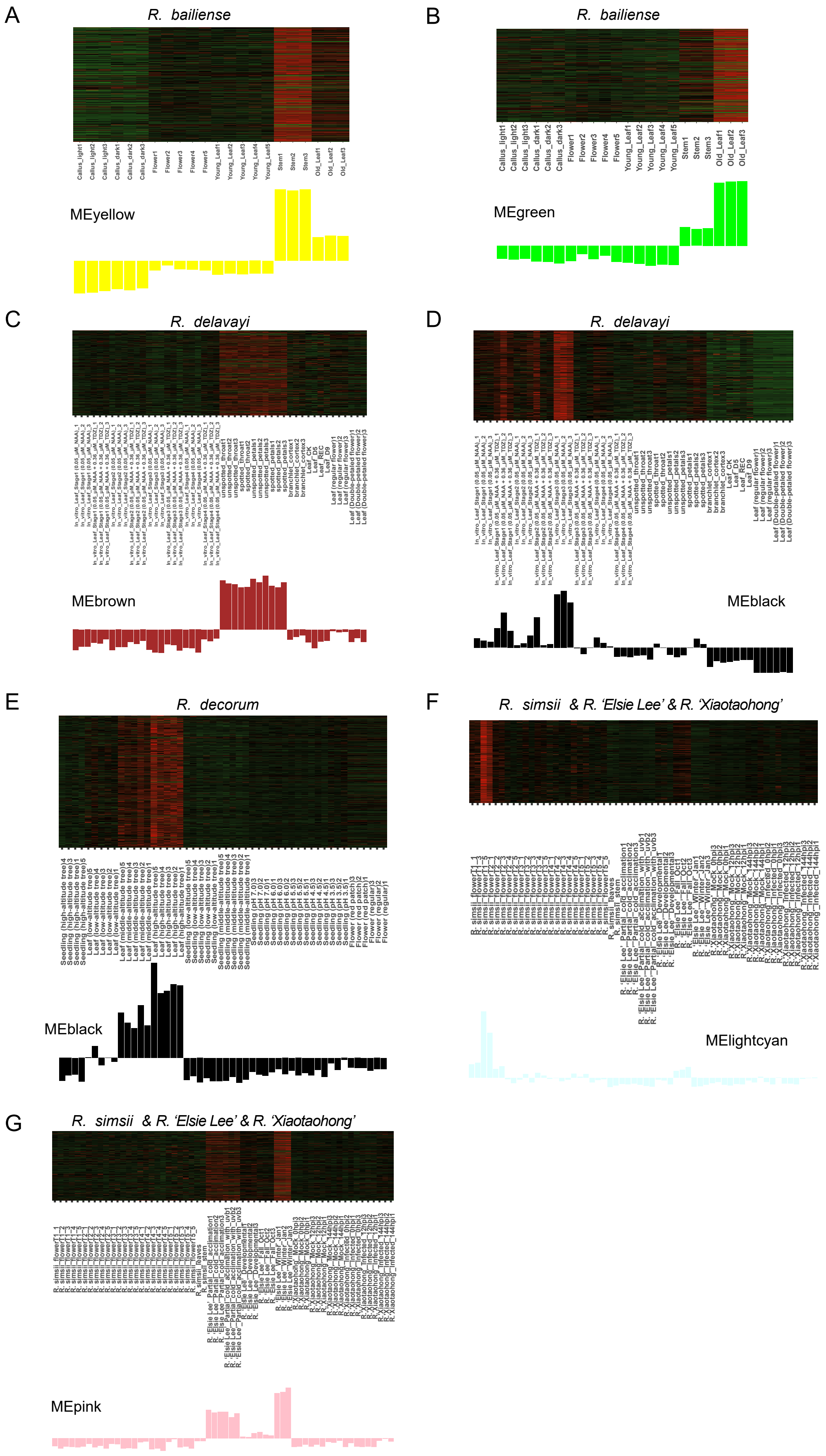

Supplementary Figure S11. Expression patterns of genes in different *Rhododendron* modules. (A-G) The gene expression heatmap illustrates the expression patterns of genes within the modules of interest: MEyellow (A) and MEgreen (B) in *R. bailiense*; MEbrown (C) and MEblack (D) in *R. delavayi*; MEblack (E) in *R. decorum*; MElightcyan (F) and MEpink (G) in *R. simsii, R. 'Elsie Lee'*, and *R. 'Xiaotaohong'*. The bar charts below the expression heatmap indicate the correlation levels of module gene expression with different plant tissues or conditions; upward indicates a positive correlation, while downward indicates a negative correlation.

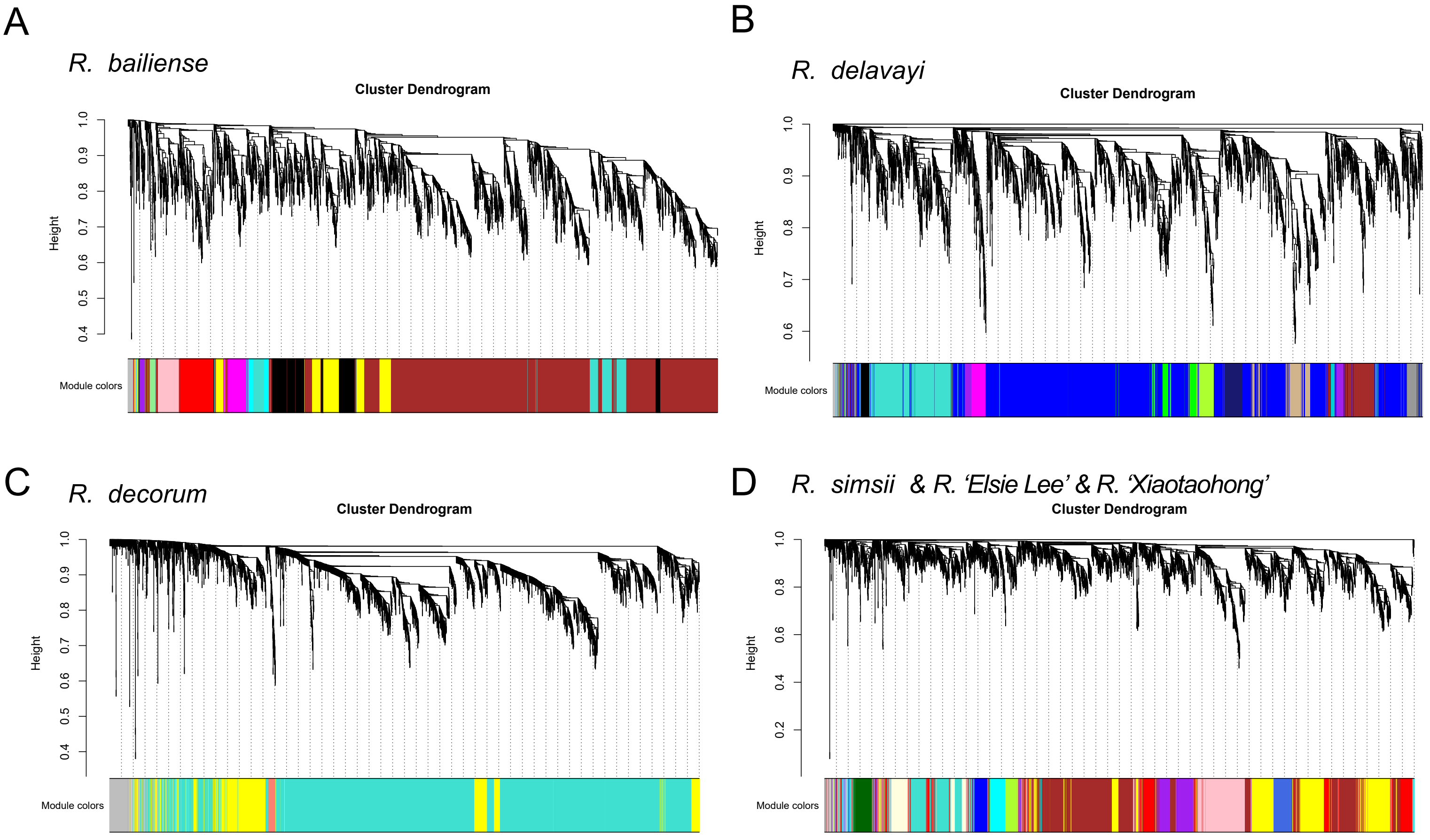

Supplementary Figure S12. Hierarchical clustering dendrograms and module divisions from WGCNA analysis of different *Rhododendron* species. (A-D) *R. bailiense* (A), *R. delavayi* (B), *R. decorum* (C), and *R. simsii* & *R. 'Elsie Lee'* & *R. 'Xiaotaohong'* (D) showing hierarchical clustering trees and module divisions of gene expression under different conditions. The parameters for module division utilized the default settings of WGCNA_shiny, with Min_Module_Size and Module_Cuttree_Height set to 30 and 0.25, respectively.

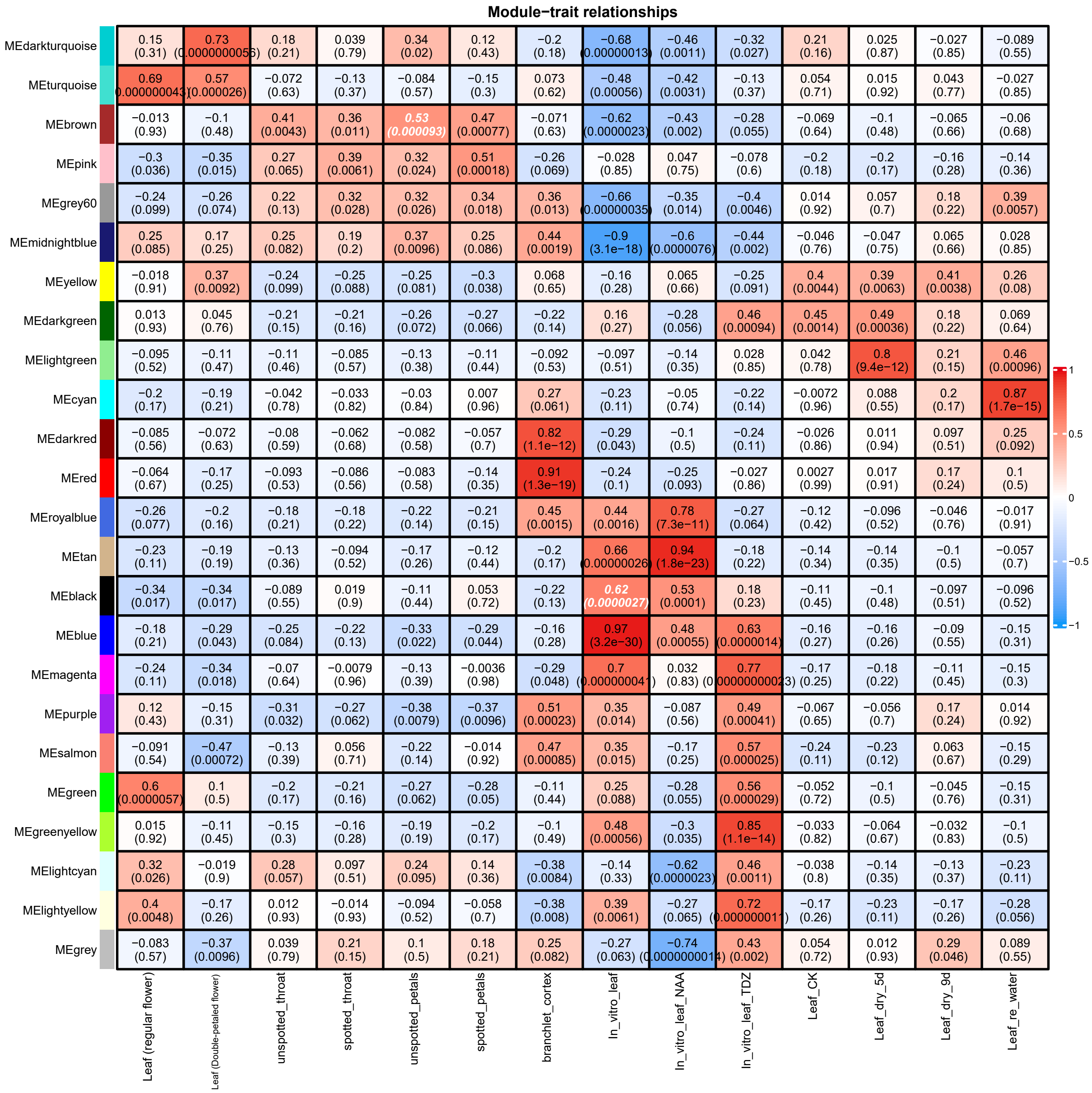

Supplementary Figure S13. The heatmap displays the correlation coefficients between modules and different tissue samples of *R. delavayi*. The correlation coefficients between the annotated modules and the samples are represented by the colors of the cells and the internal text (the Pearson correlation coefficient is shown above, and the P-value is shown below). Red and blue represent positive and negative correlations, respectively. The association values of MEbrown (containing *Rhdel03G0341700*, CAP-b) with Unspotted Petals, and MEblack (containing *Rhdel08G0101000*, CAP-d) with *In Vitro* Leaf are notably high, highlighted in italic white font.

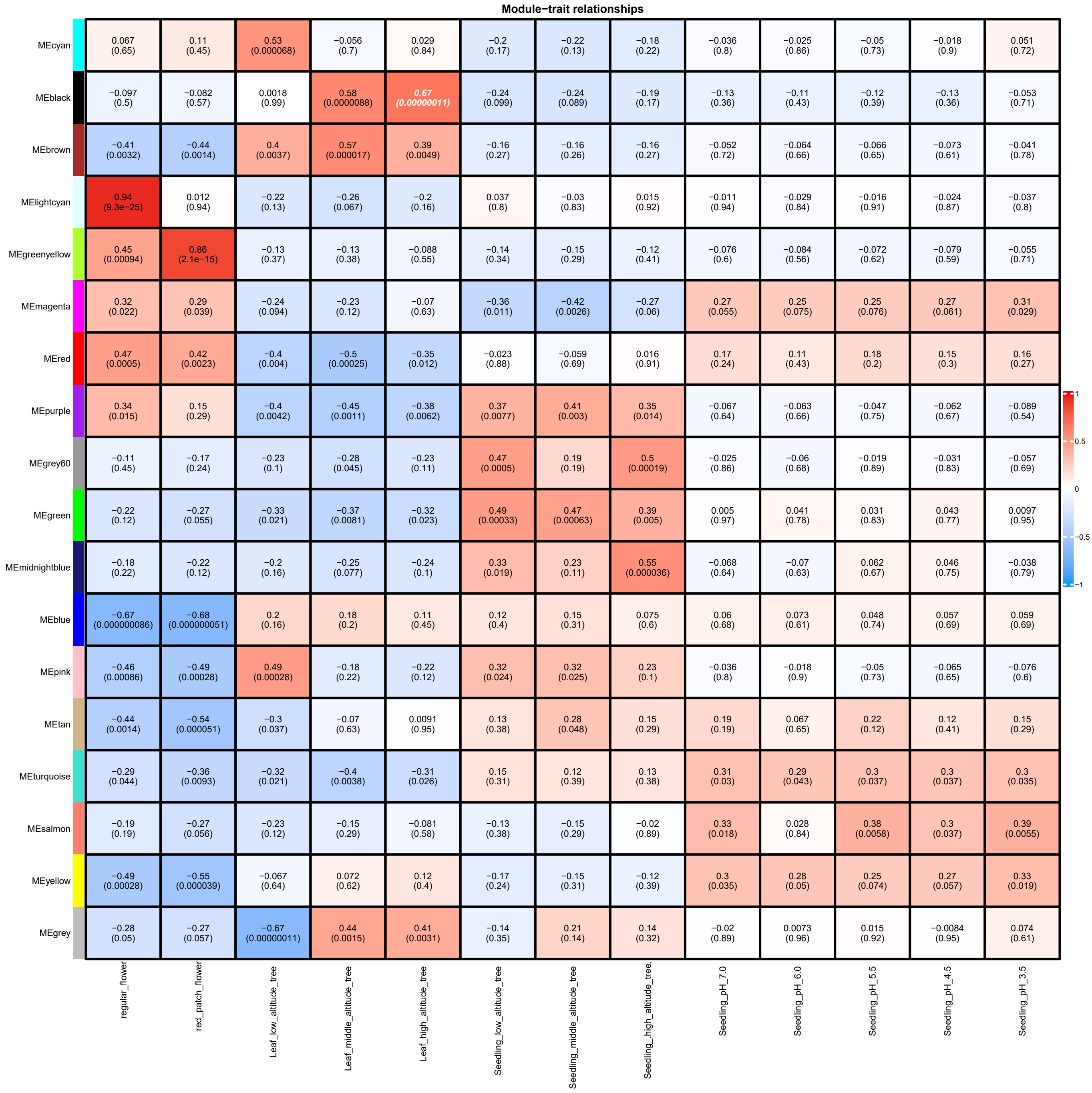

Supplementary Figure S14. The heatmap displays the correlation coefficients between modules and different tissue samples of *R. decorum*. The correlation coefficients between modules and samples are indicated by the colors of the cells and the internal text (with the Pearson correlation coefficient displayed above and the P-value below). Red and blue represent positive and negative correlations, respectively. The association values for MEblack (containing *Rde03G034180*, CAP-b; *Rde08G009950*, CAP-d) with Leaf (high altitude tree) are notably high and highlighted in italicized white text.

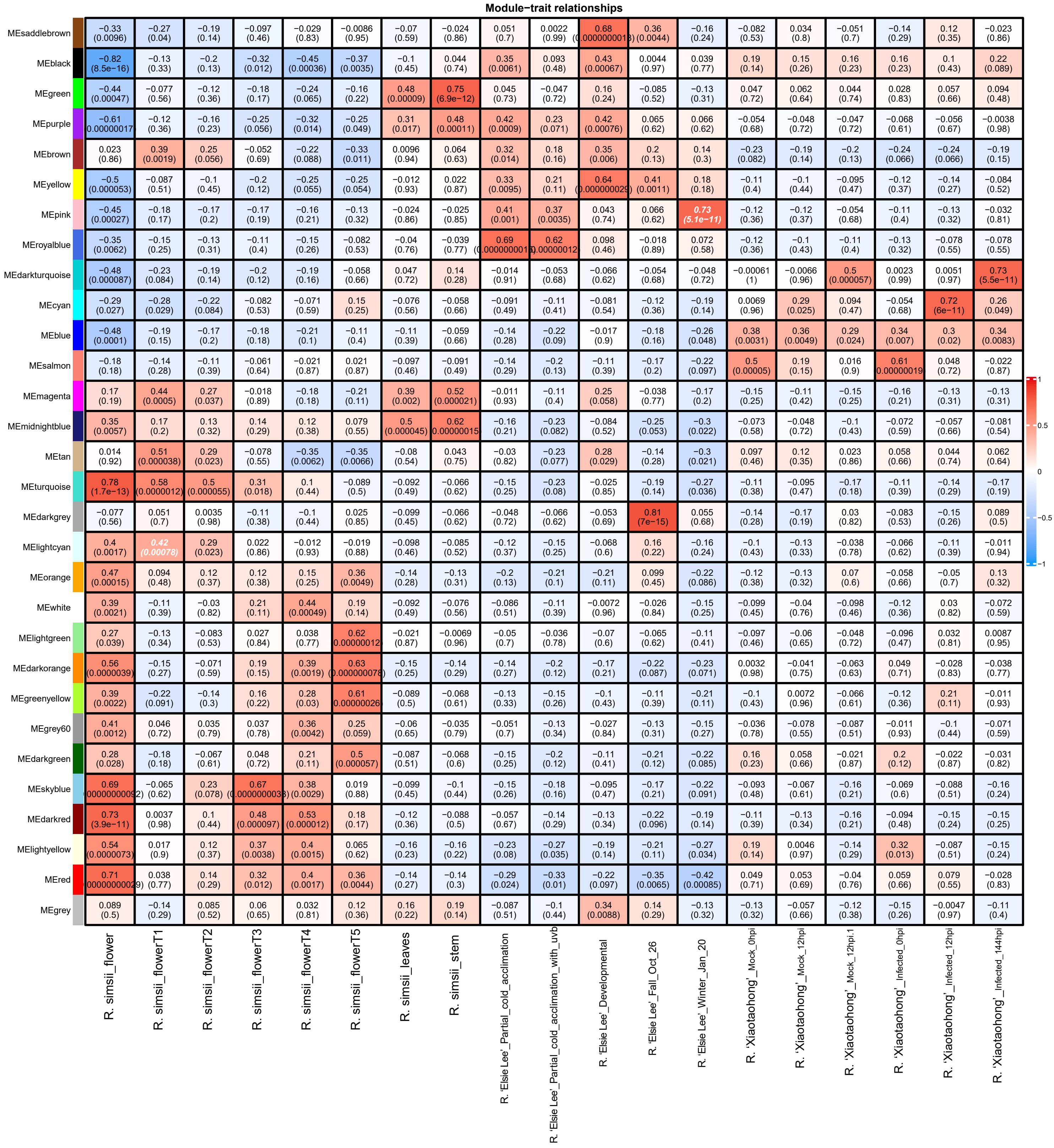

Supplementary Figure S15. The heatmap displays the correlation coefficients between modules and different tissue samples of *R. simsii, R. 'Elsie Lee'*, and *R. 'Xiaotaohong'*. The correlation coefficients between the annotated modules and samples are represented by the color of the cells and the internal text (with the Pearson correlation coefficient displayed above and the P-value below). Red and blue represent positive and negative correlations, respectively. The association values of MEpink (containing *Rhsim03G0267700*, CAP-b) corresponding to R. 'Elsie Lee' Leaf (Winter) and MElightcyan (containing *Rhsim08G0094100*, CAP-d) corresponding to *R. simsii* Flower (T1) are notably high and are emphasized in white italic font.

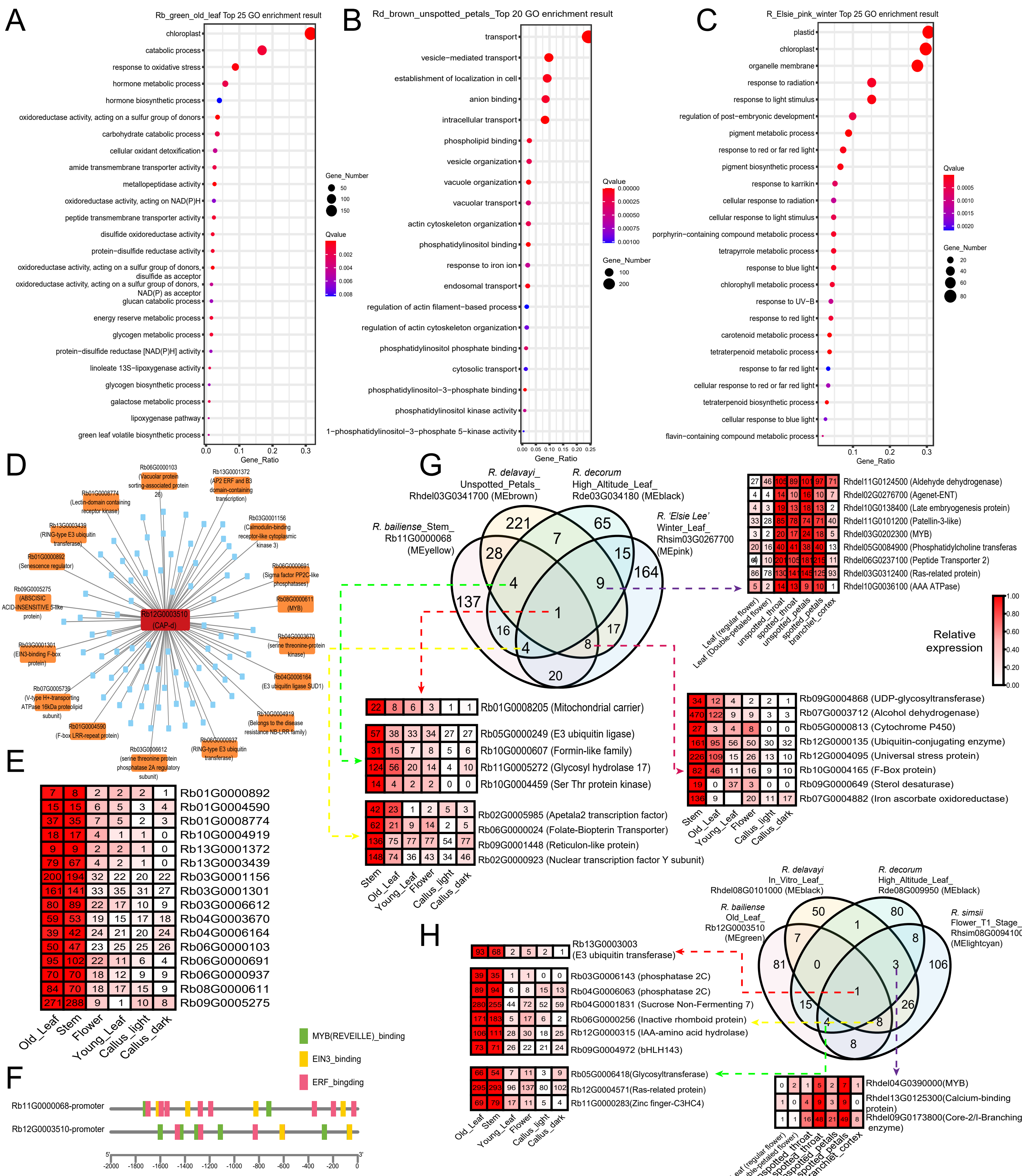

Supplementary Figure S16. Advanced analysis of co-expression networks of CAPs in various *Rhododendron* species. (A) GO functional enrichment analysis of genes in MEgreen from *R. bailiense*, significantly positively correlated with Old Leaf, containing *Rb12G0003510* (CAP-d). (B) GO functional enrichment analysis of genes in MEbrown from *R. delavayi*, significantly positively correlated with Unspotted Petals, containing *Rhdel03G0341700* (CAP-b). (C) GO functional enrichment analysis of genes in MEpink from *R. 'Elsie Lee'*, significantly positively correlated with Winter Leaves, containing *Rhsim03G0267700* (CAP-b). (D) Co-expression network closely associated with *Rb12G0003510*. Genes selected for their potential important functions and key connections are indicated by orange squares, with gene names and loci labeled. (E) The expression patterns of the selected potential key co-expressed genes. (F) A schematic diagram of the MYB (REVEILLE), EIN3, and ERF binding sites in the promoter regions of *Rb11G0000068* (CAPb1) and *Rb12G0003510* (CAPd1). (G and H) Venn diagrams illustrate the commonalities and differences among co-expressed genes of various CAP-b and d genes, and the heatmap displays the expression patterns of common genes in *R. bailiense* and *R. delavayi*. We constructed the Venn diagrams based on the commonality or differences of "gene names" to identify conserved co-expressed genes present during the high expression of CAP-b or d in rhododendrons. This may suggest the presence of some critical genes. The values in the squares indicate the FPKM values of gene expression.

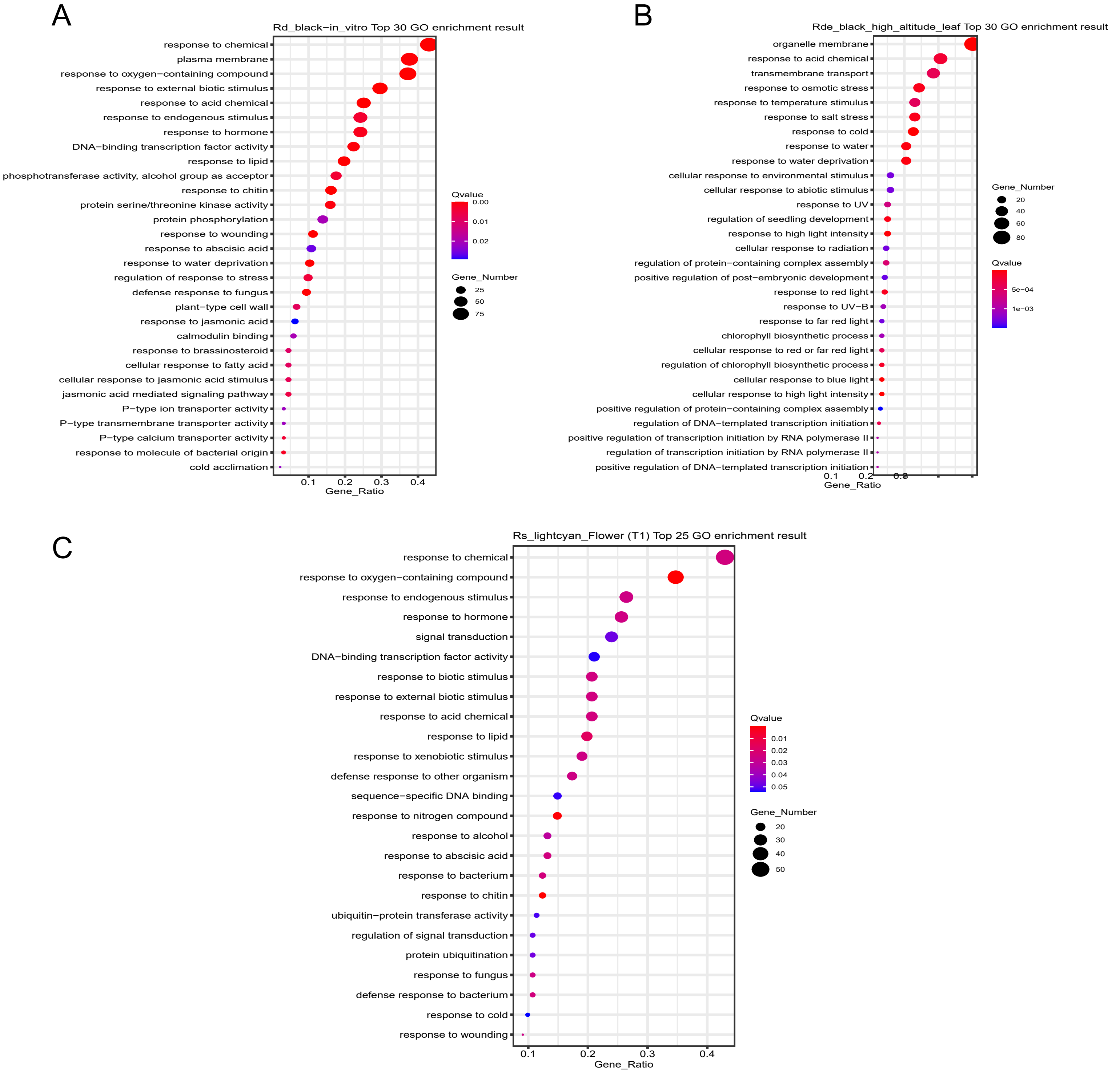

Supplementary Figure S17. Gene functional enrichment analysis of modules of additional interest in the three rhododendrons. (A) GO functional enrichment analysis of genes in MEblack (significantly positively correlated with *In Vitro* Leaf, containing *Rhdel08G0101000* (CAP-d)) in *R. delavayi*. (B) GO functional enrichment analysis of genes in MEblack (significantly positively correlated with Leaf (high altitude tree), containing *Rde03G034180* (CAP-b) and *Rde08G009950* (CAP-d)) in *R. decorum*. (C) GO functional enrichment analysis of genes in MElightcyan (significantly positively correlated with Flower (T1 stage), containing *Rhsim08G0094100* (CAP-d)) in *R. simsii*. For the original data of functional enrichment analysis, please refer to Supplementary Data Set S2.

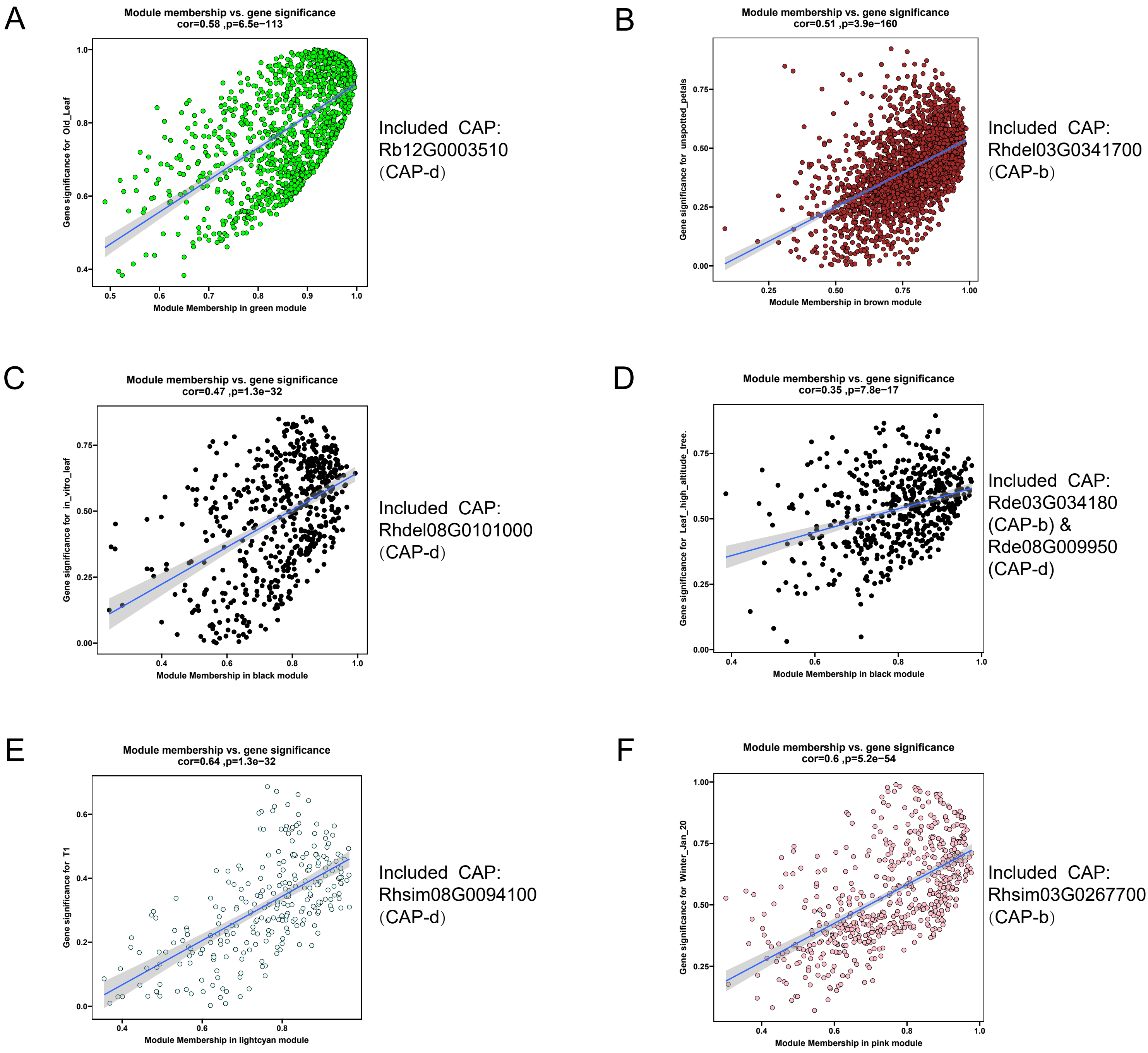

Supplementary Figure S18. The association between Module membership and Gene significance for other modules containing CAPs. (A-F): The scatter plots illustrate the associations between Module membership and Gene significance for the following modules under different conditions or tissues: MEgreen in *R. bailiense* with Old Leaf (A), MEbrown in *R. delavayi* with Unspotted Petals (B), MEblack in *R. delavayi* with *In Vitro* Leaf (C), MEblack in *R. decorum* with Leaf (high altitude tree) (D), MElightcyan in *R. simsii* with Flower (T1 stage) (E), and MEpink in *R. 'Elsie Lee'* with Leaf (Winter) (F).

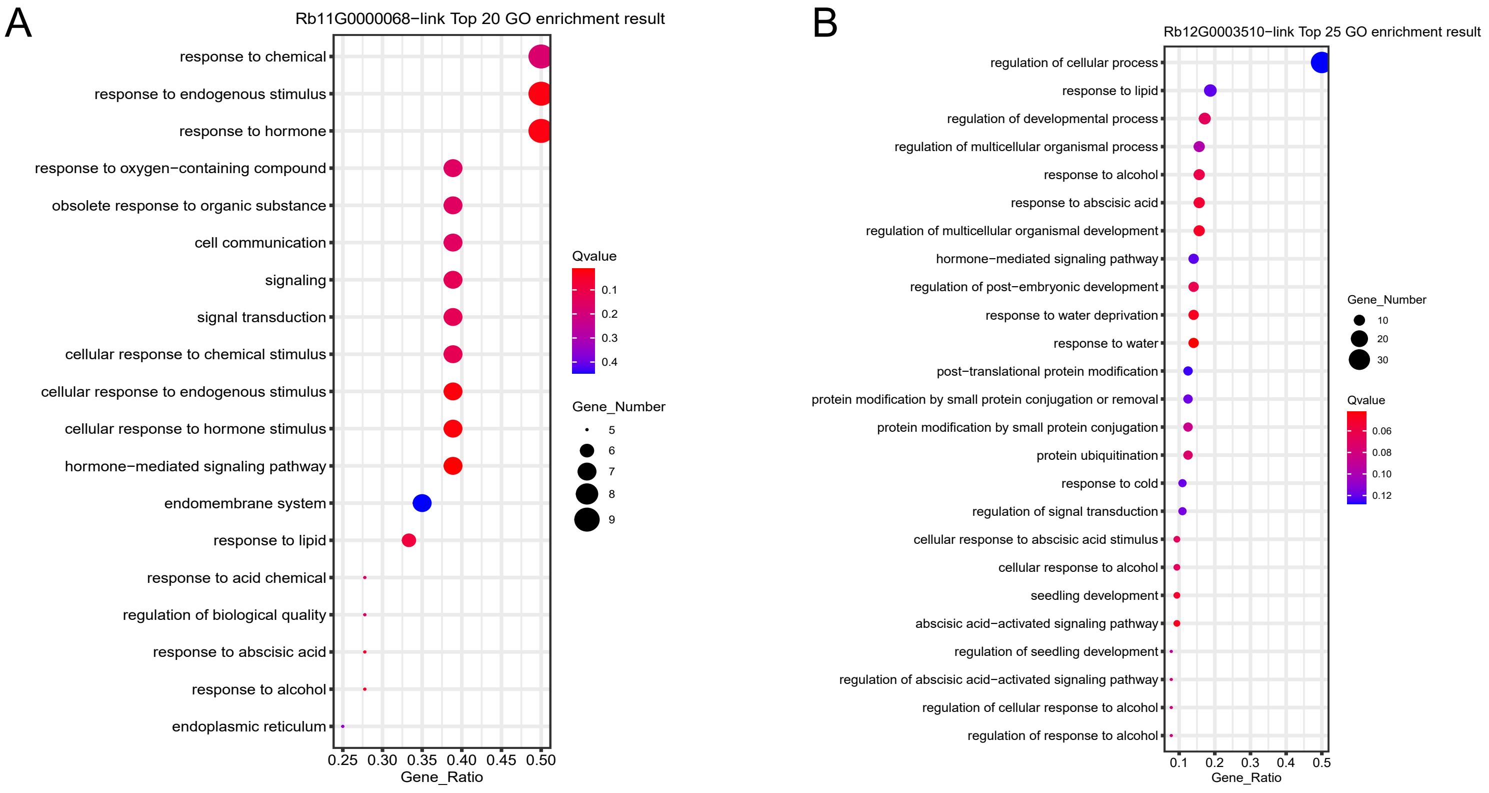

Supplementary Figure S19. Co-expression gene enrichment analysis of CAP-b and d in *R. bailiense*. (A and B) GO enrichment analysis of co-expressed genes *Rb11G0000068* and *Rb12G0003510*. The enrichment analysis includes GO terms categorized under "Molecular Function" and "Biological Process ." Only GO terms with a level greater than 3 and those that are significantly enriched are presented, while some redundant and outdated terms are excluded.

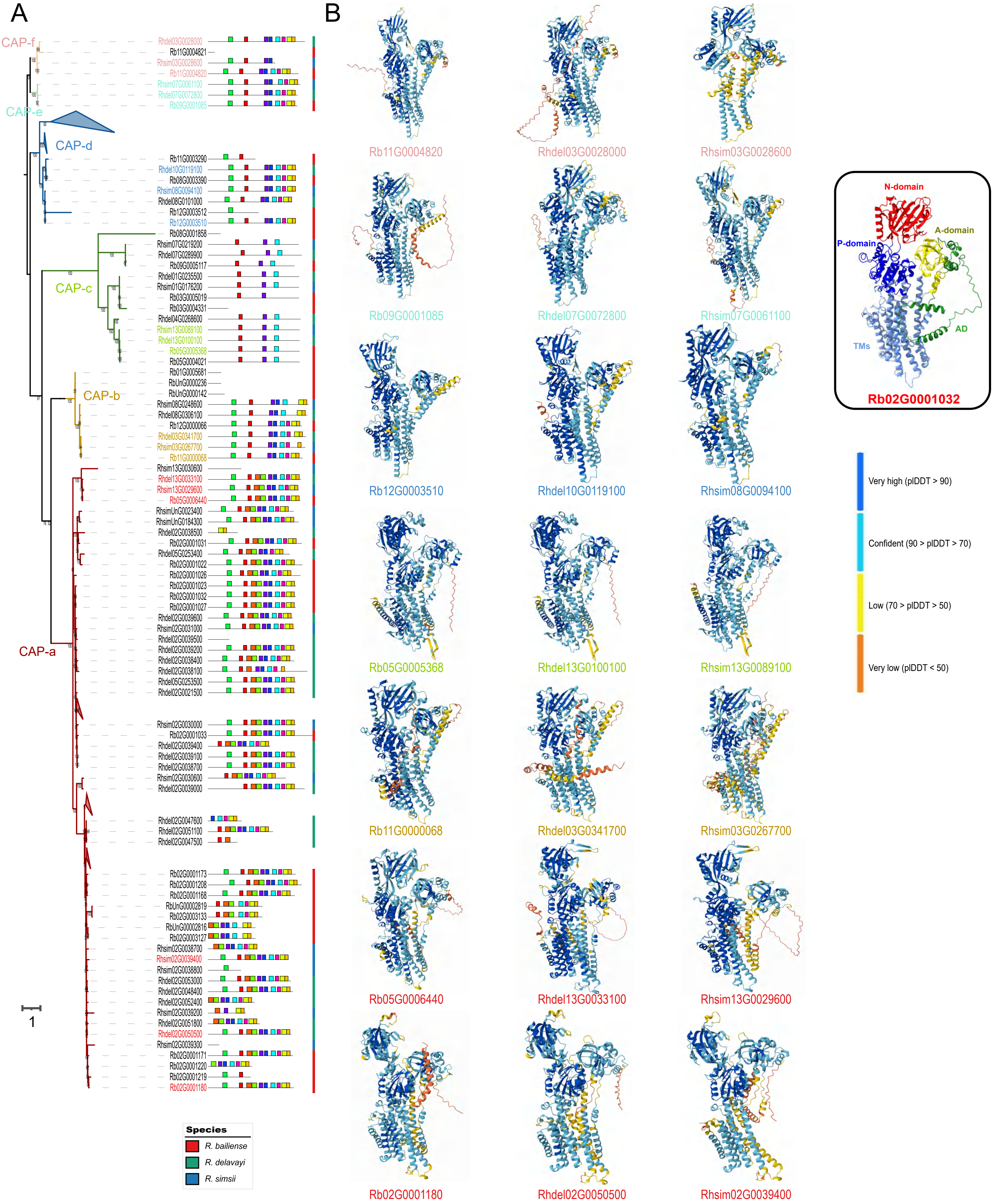

Supplementary Figure S20. Three-dimensional structural characteristics of CAPs. (A) The phylogenetic tree of CAPs from *R. bailiense*, *R. delavayi*, and *R. simsii* illustrates the protein clustering and their conserved motifs. The scale bar represents the mean number of amino acid substitutions per site. (B) Three-dimensional structure predictions for representative CAP-a to f members. The structural features of CAPs exhibit a high degree of conservation. The inset on the right is a schematic diagram of the functional domain partitioning for the representative *Rb02G0001032*. The color of the names indicates the corresponding subfamily, which can be located in (A) for evolutionary position. The structural prediction tool used is AlphaFold3. The color of the structures represents the pLDDT values. Structures with the majority of pLDDT values above 70 are considered to be at a confident level.

Supplementary Table S1. Sources of data resources for this study.

Genome

Species	Resource
Actinidia chinensis	https://ngdc.cncb.ac.cn/gwh/ncbi_assembly/55381/show
Amborella trichopoda	https://ngdc.cncb.ac.cn/gwh/Assembly/92602/show
Arabidopsis thaliana	https://www.ncbi.nlm.nih.gov/datasets/genome/GCF_000001735.4/
Asparagus officinalis	https://ngdc.cncb.ac.cn/gwh/ncbi_assembly/55199/show
Camellia sinensis	https://ngdc.cncb.ac.cn/gwh/ncbi_assembly/1663139/show
Camptotheca acuminata	https://ngdc.cncb.ac.cn/gwh/ncbi_assembly/2446564/show
Candidatus Lokiarchaeum	https://ngdc.cncb.ac.cn/gwh/ncbi_assembly/353/show
Chara braunii	https://ngdc.cncb.ac.cn/gwh/ncbi_assembly/55509/show
Chlamydomonas reinhardtii	https://ngdc.cncb.ac.cn/gwh/ncbi_assembly/55329/show
Coffea canephora	https://ngdc.cncb.ac.cn/gwh/ncbi_assembly/55704/show
Daucus carota	https://ngdc.cncb.ac.cn/gwh/ncbi_assembly/2404622/show
Diphasiastrum complanatum	https://ngdc.cncb.ac.cn/gwh/ncbi_assembly/57886/show
Dunaliella salina	https://ngdc.cncb.ac.cn/gwh/ncbi_assembly/56241/show
Ginkgo biloba	https://ngdc.cncb.ac.cn/gwh/Assembly/18742/show
Glycine max	https://ngdc.cncb.ac.cn/gwh/ncbi_assembly/56502/show
Helianthus annuus	https://ngdc.cncb.ac.cn/gwh/ncbi_assembly/3063396/show
Marchantia polymorpha	https://ngdc.cncb.ac.cn/gwh/ncbi_assembly/2307591/show
Nymphaea colorata	https://ngdc.cncb.ac.cn/gwh/Assembly/651/show
Oryza sativa	https://www.ncbi.nlm.nih.gov/datasets/genome/GCF_034140825.1/
Ostreococcus lucimarinus	https://ngdc.cncb.ac.cn/gwh/ncbi_assembly/54980/show
Physcomitrella patens	https://ngdc.cncb.ac.cn/gwh/Assembly/88054/show
Porphyra umbilicalis	https://ngdc.cncb.ac.cn/gwh/ncbi_assembly/55301/show
Rhododendron bailiense	https://ngdc.cncb.ac.cn/gwh/Assembly/83793/show
Rhododendron decorum	http://www.tegr.com.cn/Download-Sequence
Rhododendron delavayi	http://bioinfor.kib.ac.cn/RPGD/download_genome.html
Rhododendron griersonianum	www.ncbi.nlm.nih.gov/datasets/genome/GCA_018127125.1
Rhododendron henanense	https://ngdc.cncb.ac.cn/gwh/Assembly/22219/show
Rhododendron irroratum	http://bioinfor.kib.ac.cn/RPGD/download_genome.html
Rhododendron liliiflorum	http://www.tegr.com.cn/Download-Sequence
Rhododendron molle	https://ngdc.cncb.ac.cn/gwh/Assembly/29302/show
Rhododendron ovatum	http://bioinfor.kib.ac.cn/RPGD/download_genome.html
Rhododendron prattii	https://ngdc.cncb.ac.cn/gwh/Assembly/24363/show
Rhododendron ripense	https://plantgarden.jp/en/download/t224351/t224351.G001/
Rhododendron simsii	www.ncbi.nlm.nih.gov/datasets/genome/GCA_014282245.1
Rhododendron vialii	https://www.ncbi.nlm.nih.gov/datasets/genome/GCF_030253575.1/
Rhododendron williamsianum	http://bioinfor.kib.ac.cn/RPGD/download_genome.html
Selaginella moellendorffii	https://ngdc.cncb.ac.cn/gwh/ncbi_assembly/55382/show
Sesamum indicum	https://ngdc.cncb.ac.cn/gwh/ncbi_assembly/54903/show
Solanum lycopersicum	https://phytozome-next.jgi.doe.gov/
Theobroma cacao	https://ngdc.cncb.ac.cn/gwh/ncbi_assembly/55153/show
Vaccinium myrtillus	http://www.tegr.com.cn/Download-Sequence
Vitis vinifera	https://ngdc.cncb.ac.cn/gwh/ncbi_assembly/55007/show
Zea mays	https://ngdc.cncb.ac.cn/gwh/ncbi_assembly/56109/show

**Transcriptome**	
Species	NCBI-Bioproject
Rhododendron agastum	PRJNA1016934 (Newly sequenced)
Rhododendron bailiense	PRJNA949618, PRJNA1018501
Rhododendron decorum	PRJNA1018084 (Newly sequenced), PRJNA774925 (Public),
Rhododendron delavayi	PRJNA1278900 (Newly sequenced), PRJNA816456 (Public),
Rhododendron 'Elsie Lee'	PRJNA574773 (Public)
Rhododendron griersonianum	PRJNA798217 (Public)
Rhododendron molle	PRJNA804375 (Public)
Rhododendron simsii	PRJNA588298 (Public)
Rhododendron 'Xiaotaohong'	PRJNA940189 (Public)
Rhododendron ×pulchrum	PRJNA858916 (Public)
**Genome resequencing**	
Species	NCBI-Bioproject
Rhododendron delavayi	PRJNA659608

Supplement Table S2. Primers used in this study.

Genes	Primers
CAPb1-Rb11G0000068-F	gattctagaactagtATGGAAACAGCGAATGACGAATTCAA
CAPb1-Rb11G0000068-R	tgactcgaggtcgacCTATTTTCTCTCAACACCATCCTCCTGC
CAPd1-Rb12G0003510-F	gattctagaactagtATGGAGAGCTTGCTAGATGAGAACTTT
CAPd1-Rb12G0003510-R	tgactcgaggtcgacTCATGTATTCACAGGTATCATCTTCAAGCC
CIPK1-Rb01G0008246-F	gattctagaactagtATGGTAGGGTGGTTTTCTGGGGA
CIPK1-Rb01G0008246-R	tgactcgaggtcgacTCAAACTTCCATGGTCATCAGCCC
CIPK2-Rb03G0000553-F	gattctagaactagtATGGCGGATAAAGGCGAGAAAT
CIPK2-Rb03G0000553-R	tgactcgaggtcgacTCAACTCTCAACAGCAGGGGA
CIPK3-Rb04G0000153-F	gattctagaactagtATGCCGGAAATTAAGGATGATTCCG
CIPK3-Rb04G0000153-R	tgactcgaggtcgacTTAACTACCGGTAACCCGCCG
CBL1-Rb09G0002570-F	gattctagaactagtATGGATCCAAGAAGCAACTCATCG
CBL1-Rb09G0002570-R	tgactcgaggtcgacTCAGTCTTCAACCACGGTGTTG
GAP-pro-F	tttgcggccggtaccTCATTATCAATACTCGCCATTTCAAAGAATACG
CYC1-ter-R	ggcgaattgggtaccGCAAATTAAAGCCTTCGAGCGTCC
